# Perturbation of serine enantiomers homeostasis in the striatum of MPTP-lesioned monkeys and mice reflects the extent of dopaminergic midbrain degeneration

**DOI:** 10.1016/j.nbd.2023.106226

**Published:** 2023-07-13

**Authors:** Marcello Serra, Anna Di Maio, Valentina Bassareo, Tommaso Nuzzo, Francesco Errico, Federica Servillo, Mario Capasso, Pathik Parekh, Qin Li, Marie-Laure Thiolat, Erwan Bezard, Paolo Calabresi, David Sulzer, Manolo Carta, Micaela Morelli, Alessandro Usiello

**Affiliations:** a Department of Biomedical Sciences, University of Cagliari, Monserrato, Italy; b Laboratory of Translational Neuroscience, CEINGE Biotecnologie Avanzate Francesco Salvatore, Naples, Italy; c Department of Environmental, Biological and Pharmaceutical Science and Technologies, Università Degli Studi della Campania “Luigi Vanvitelli“, Caserta, Italy; d Department of Agricultural Sciences, University of Naples “Federico II”, Naples, Italy; e Department of Neuroscience, Cattolica Sacro Cuore University, Rome, Italy; f Dipartimento di Medicina Molecolare e Biotecnologie Mediche, Università degli Studi di Napoli Federico II, Via Pansini, 5, Napoli 80131, Italy; g Motac Neuroscience, UKM15 6WE, Manchester, United Kingdom; h Institute of Lab Animal Sciences, China Academy of Medical Sciences, Beijing, China; i Université de Bordeaux, Institut des Maladies Neurodégénératives, Bordeaux, France; j Centre National de la Recherche Scientifique Unité Mixte de Recherche 5293, Institut des Maladies Neurodégénératives, Bordeaux, France; k Neurologia, Policlinico Universitario A. Gemelli, IRCCS, Rome, Italy; l Departments of Psychiatry, Neurology, Pharmacology, Columbia University Irving Medical Center, Division of Molecular Therapeutics, New York State Psychiatric Institute, New York, NY 10032, USA; m National Research Council of Italy, Institute of Neuroscience, Cagliari, Italy

**Keywords:** Parkinson’s disease, caudate putamen, serine racemase, alanine serine cysteine transporter 1 (ASCT1), 3-phosphoglycerate dehydrogenase (PHGDH), ionotropic *N*-methyl-d-aspartate receptors, (NMDAR), excitatory amino acids, synaptic plasticity, neuroinflammation

## Abstract

Loss of dopaminergic midbrain neurons perturbs l-serine and d-serine homeostasis in the *post-mortem* caudate putamen (CPu) of Parkinson’s disease (PD) patients. However, it is unclear whether the severity of dopaminergic nigrostriatal degeneration plays a role in deregulating serine enantiomers’ metabolism. Here, through high-performance liquid chromatography (HPLC), we measured the levels of these amino acids in 1-methyl-4-phenyl-1,2,3,6-tetrahydropyridine (MPTP)-treated monkeys and MPTP-plus-probenecid (MPTPp)-treated mice to determine whether and how dopaminergic midbrain degeneration affects the levels of serine enantiomers in various basal ganglia subregions. In addition, in the same brain regions, we measured the levels of key neuro-active amino acids modulating glutamatergic neurotransmission, including l-glutamate, glycine, l-aspartate, d-aspartate, and their precursors l-glutamine, l-asparagine. In monkeys, MPTP treatment produced severe denervation of nigrostriatal dopaminergic fibers (~75%) and increased the levels of serine enantiomers in the rostral putamen (rPut), but not in the subthalamic nucleus, and the lateral and medial portion of the globus pallidus. Moreover, this neurotoxin significantly reduced the protein expression of the astrocytic serine transporter ASCT1 and the glycolytic enzyme GAPDH in the rPut of monkeys. Conversely, concentrations of d-serine and l-serine, as well as ASCT1 and GAPDH expression were unaffected in the striatum of MPTPp-treated mice, which showed only mild dopaminergic degeneration (~30%). These findings unveil a link between the severity of dopaminergic nigrostriatal degeneration and striatal serine enantiomers concentration, ASCT1 and GAPDH expression. We hypothesize that the up-regulation of d-serine and l-serine levels occurs as a secondary response within a homeostatic loop to support the metabolic and neurotransmission demands imposed by the degeneration of dopaminergic neurons.

## 1. Introduction

Parkinson’s disease (PD) is a progressive neurodegenerative disorder characterized by alpha-synuclein accumulation and the loss of dopaminergic neurons in the substantia nigra pars compacta (SNc), leading to the progressive manifestation of motor and non-motor symptoms (Obeso et al., 2017). Although PD is considered a disorder primarily affecting the dopaminergic system, evidence suggests that other neurotransmitter systems also contribute to the appearance of the disease features. Among these, molecular alterations occurring at glutamatergic synapses, and in particular at the ionotropic *N*-methyl-d-aspartate (NMDA) receptors (NMDAR), were suggested to play a pivotal role (Campanelli et al., 2022). In fact, over the past two decades, extensive research in PD patients and experimental animal models has provided evidence of significant structural and functional alterations impacting NMDAR signaling (Gardoni and Di Luca, 2015; Hallett et al., 2005; Picconi et al., 2012). However, our understanding of presynaptic events, including the levels of excitatory neurotransmitters that regulate the activation of these receptors, remains limited.

Previous studies found altered concentrations of amino acids involved in the modulation of glutamatergic transmission in the cerebrospinal fluid (CSF) and brain of PD patients (Jiménez-Jiménez et al., 2020; Kish et al., 1986; Nuzzo et al., 2019; Rinne et al., 1988). Very recently, our group reported that the severe loss of dopaminergic midbrain neurons is correlated with significant elevation in serine enantiomers levels and downregulation of the astrocytic alanine serine cysteine transporter 1 (ASCT1), also known as SLC1A4 (Hofmann et al., 1994), in *post-mortem* caudate putamen (CPu) of PD patients with different Braak Lewy Body stage severity (3-4 and 6) compared to non-demented controls (Di Maio et al., 2023).

Whereas abnormally high levels of d-serine appear to produce detrimental effects in ALS (Kondori et al., 2017; Sasabe et al., 2012; Sasabe et al., 2007; Thompson et al., 2012) and in animals following a traumatic brain injury (TBI) (Perez et al., 2017; Tapanes et al., 2022), this does not appear to be the case for PD. Indeed, preclinical research conducted in dopamine (DA)-depleted rodents and primates provided significant evidence supporting the beneficial effects of direct and indirect agonists of the glycine site of NMDAR in the treatment of PD (Frouni et al., 2022; Frouni et al., 2021; Ho et al., 2011; Schmitz et al., 2013; Schneider et al., 2000; Zhang et al., 2021). In agreement with these observations, promising results were obtained in a small double-blind clinical study designed to evaluate the therapeutic application of oral d-serine supplementation as an adjunctive treatment to standard PD medications (Gelfin et al., 2012).

Although not directly involved in NMDAR occupancy, l-serine acts as a precursor for the synthesis of the endogenous NMDAR co-agonists, glycine and d-serine (Metcalf et al., 2018; Takano et al., 2016). Consequently, the levels of l-serine in the brain can influence NMDAR-dependent synaptic plasticity and spatial memory, as recently observed in an animal model of AD (Le Douce et al., 2020).

Consistent with this, in the present study, we took advantage of two well-established preclinical models of PD characterized by different degrees of dopaminergic nigrostriatal degeneration to assess whether the severity of dopaminergic loss may affect the striatal levels of serine enantiomers levels. We utilized the MPTP *Macaca Mulatta* model of PD, in which parkinsonian motor deficits are associated with the severe loss of dopaminergic nigrostriatal neurons (Engeln et al., 2015; Nuzzo et al., 2019), and the sub-chronic MPTP plus probenecid (MPTPp) mouse model of PD, characterized by mild dopaminergic neurodegeneration (Baranyi et al., 2016), which is best suitable to reveal the presence of subtle neurodegenerative events.

Our findings indicate that a substantial loss of dopaminergic input in the CPu is necessary to induce detectable changes in serine enantiomer levels in the PD brain.

## 2. Materials and methods

### 2.1. Drugs

MPTP-HCl was purchased from Toronto Research Chemicals, Canada (D463595) and was dissolved in distilled water. Probenecid (P8761) and d-serine (S425) were purchased from Merck and dissolved in 5% NaHCO3 and drinking water, respectively.

### 2.2. Animals

#### 2.2.1. Non-human primates

Ten captive-bred female macaques (*Macaca mulatta*, Xierxin, Beijing, PR of China; mean age = 5 ± 1 years; mean weight = 5.3 ± 0.8kg), were housed in individual primate cages under controlled conditions of humidity (50 ± 5%), temperature (24 ± 1 °C), and light (12h light/12h dark cycles, time lights on 8:00 am), and allowing visual contacts and interaction with macaques housed in the adjacent cages. Food and water were available ad libitum and animal care was supervised daily by veterinarians skilled in the healthcare and maintenance of non-human primates. Experiments were carried out in accordance with European Communities Council Directive (2010/63/EU) for the care of laboratory animals in an AAALAC-accredited facility following acceptance of the study design by the Institute of Lab Animal Science IACUC (Chinese Academy of Medical Sciences, Beijing, China).

#### 2.2.2. C57BL/6 J mice

Fifty-seven, 12-weeks old adult male C57BL/6J mice (Charles River, Calco, Italy), weighing 25 ± 2 g at the beginning of experiments were used. Mice were housed in groups of 4/5 per cage under constant temperature (22 ± 1 °C) and a 12-h light/dark cycle, with ad libitum access to water and food. All studies were performed in accordance with the ARRIVE recommendations, the guidelines for animal experimentation of the EU directives (2010/63/EU; L.276; 22/09/2010), and with policies issued by the Organism for Animal Welfare (OPBA) of the University of Cagliari. Experiments were designed to minimize animal discomfort and reduce the number of animals used.

### 2.3. Experimental plan and tissue collection

#### 2.3.1 Monkeys

Out of ten macaques, five received daily MPTP hydrochloride injections (0.2 mg/kg, intravenously) until parkinsonian signs appeared (Bezard et al., 2020; Engeln et al., 2015; Hulme et al., 2022; Urs et al., 2015). The remaining five monkeys were used as control. Animal sacrifice and processing of tissues were carried out according to a previous protocol (Bezard et al., 2001). Briefly, all the animals were sacrificed by sodium pentobarbital overdose (150 mg/kg, i.v.), and the brains were quickly removed after death. Each brain was bisected along the midline, and the two hemispheres were immediately frozen by immersion in isopentane (−45 °C) and then stored at −80 °C. Coronal 300 μm-thick sections were cryostat-cut and punches of brain tissue were taken for the following regions: rostral putamen (rPut), subthalamic nucleus (STN), lateral and medial part of globus pallidus (GP).

#### 2.3.2. Mice

C57BL/6J mice received intraperitoneal (i.p.) injections of either saline (vehicle) or MPTP (25 mg/kg) plus probenecid (100 mg/kg, 30 min before MPTP) (MPTPp), twice a week for five consecutive weeks. Afterward, mice were subjected to seven days of drug washout, before receiving either d-serine (850 mg/L) in the drinking water or standard drinking water for four consecutive weeks. This protocol results in a daily dose of approximately 100 mg/kg of d-serine (average mouse weight: 30 g, average drinking volume: 3.5 ml/day) (Le Douce et al., 2020). Liquid consumption was recorded every 48 h, before replacing the solution with a freshly prepared one. Overall, mice were randomly allocated into four experimental groups based on the treatment, namely: vehicle/water (*n* = 10); vehicle/d-serine (*n* = 14); MPTPp/Water (*n* = 15); MPTPp/d-serine (*n* = 18). After the completion of the pharmacological treatment, mice were anesthetized before sacrifice.

Mice used for immunohistochemical evaluations were sacrificed by transcardial perfusion with cold 4% paraformaldehyde in phosphate buffer (PB, 0.1 M, pH 7.4). Afterwards, brains were removed, post-fixed in 4% paraformaldehyde for 2 h, and preserved in PB saline 1× (PBS) at 4 ° C. The next day, brains were coronally sectioned on a vibratome to obtain sections (40 μm) suited for immunohistochemical processing. For each mouse, three coronal sections were obtained according to the following stereotaxic coordinates: A) CPu: 1.34 to 0.74 mm, B) SNc: −2.92 to −3.52 mm relative to bregma.

Mice used for high-performance liquid chromatography (HPLC), and western blot (WB) evaluations were decapitated and the medial prefrontal cortex (1.98 to 1.34 mm relative to bregma), the striatum (1.34 to 0.74 relative to bregma) and the midbrain (−2.92 to −3.52 mm relative to bregma) were rapidly collected, frozen on dry ice and stored at −80 °C until use. All the brain areas were identified according to the mouse brain atlas of Paxinos and Franklin (Paxinos and Franklin, 2008).

### 2.4. High-performance liquid chromatography analysis

#### 2.4.1. Determination of brain amino acids in monkeys and mice

Brain tissue samples of monkeys and mice were homogenized in 1:20 (*w/v*) 0.2 M trichloroacetic acid (TCA), sonicated (3 cycles, 10 s each), and centrifuged at 13,000 ×*g* for 20 min. All precipitated protein pellets from brain samples were stored at −80 °C for protein quantification. The supernatants were neutralized with 0.2 M NaOH and subjected to pre-column derivatization with o-phthaldialdehyde/*N*-acetyl-l-cysteine in 50% methanol. Diastereoisomer derivatives were resolved on a UHPLC Nexera X3 system (Shimadzu) by using a Shim-pack GIST C18 3-μm reversed-phase column (Shimadzu, 4.0 × 150 mm) under isocratic conditions (0.1 M sodium acetate buffer, pH 6.2, 1% tetrahydrofuran, and 1 ml/min flow rate). A washing step in 0.1 M sodium acetate buffer, 3% tetrahydrofuran, and 47% acetonitrile was performed after every single run. Identification and quantification of amino acids were based on retention times and peak areas compared with those associated with external standards. The total protein content of brain tissue homogenates was determined by the Bradford assay method, after resolubilization of the TCA-precipitated protein pellets in 1% SDS solution. The detected amino acids concentration in tissue homogenates was normalized by the total protein content and expressed as nmol/mg protein.

#### 2.4.2. Determination of molecules involved in dopamine synthesis and metabolism in mice

Frozen striatal samples of mice were added with 200 μl of 0.2 N perchloric acid. Tissues were homogenized with ultrasounds and subsequently centrifuged at 11,000 ×*g* for 10 min at 4 °C. The supernatant was transferred into Spin-X microcentrifuge filters (0.22 μm nylon filter), centrifuged at 11000 ×*g* for 5 min at 4 °C and then stored at −80 °C until HPLC analysis. 20 μl samples (5 μl of hydrophilic phase +15 μl of 0.2 N PCA) were injected without purification into an HPLC equipped with a reverse phase column (LC-18 dB, 15 cm, 5 μm particle size, Supelco, Waters, Milford, MA, USA) and a coulometric detector (ESA, Coulochem II, Bedford, MA, USA) to quantify molecules involved in DA signaling, specifically: L-DOPA, DA, HVA, DOPAC. To detect catecholamines, their precursors, and metabolites, the first electrode of the detector was set at +125 mV (oxidation) and the second at −175 mV (reduction). The composition of the mobile phase was (in mM): 50 NaH2PO4, 0.1 Na2-EDTA, 0.5 n-octyl sodium sulfate, 15% (*v/v*) methanol, pH 3.7. The detected molecules concentration in tissue homogenates was normalized by the total tissue content expressed as femtomoles/mg of tissue (Vargiu et al., 2021).

### 2.5. Western blotting

Frozen, powdered samples from different brain regions of monkeys and mice were sonicated in 1% sodium dodecyl sulfate and boiled for 10 min. Aliquots (2 μl) of the homogenate were used for protein determination using a Bio-Rad Protein Assay kit. Equal amounts of total proteins (30 μg) for each sample were loaded on precast 4% to 20% gradient gels (BioRad Laboratories, Hercules, CA, USA), separated by sodium dodecyl sulfate–polyacrylamide gel electrophoresis, and transferred to PVDF membranes (GE Healthcare, Chicago, IL, USA) via the Trans-Blot Turbo System (BioRad Laboratories). For both monkeys and mice, immunodetections were accomplished by using the following antibodies: tyrosine hydroxylase (TH) (1:2000, MAB318, Merck), SR (1:500, sc-5751; Santa Cruz), DAAO (1:1000, EB11100; Everest Biotech), β-actin (1:10000, A5441; Merk Sigma), 3-phosphoglycerate dehydrogenase (PHGDH) (1:1000, 13428S; Cell Signaling), ASCT1 (1:1000, 8442; Cell Signaling), and glyceraldehyde-3-phosphate dehydrogenase (GAPDH) (1:1000, sc-32233; Santa Cruz Biotechnology). Blots were then incubated with α-rabbit, α-mouse, or α-goat horseradish peroxidase-conjugated secondary antibodies. Immunoreactivity was detected by enhanced chemiluminescence (GE-Healthcare) and quantified by Quantity One software (Bio-Rad). Optical density values of all markers analysed were normalized to either β-actin or GAPDH for variation in loading and transfer. Data are reported as medians, along with the interquartile range (first-third quartiles—IQR). All representative blots shown in the figures arise from cut-out and pasted bands for reassembling the image.

### 2.6. Immunohistochemical analysis (mice)

#### 2.6.1. Reaction protocol

The diaminobenzidine technique was used for the visualization of TH in the SNc, as previously described (Costa et al., 2014). In brief, free-floating sections were rinsed three times in PBS 1× and incubated with 1% H_2_O_2_ (30% *v/v*, Merck) in PBS at room temperature (10 min), to block endogenous peroxidase activity. Then, sections were blocked and permeabilized with 5% normal goat serum and 0.1% Triton X-100 at room temperature (20 min), and later incubated for 48 h (4 °C) with the primary rabbit anti-TH antibody (1:1000, Millipore, US, AB152). Thereafter, the biotinylated secondary antibody (1:500, goat anti-mouse IgG, Vector, UK) was added (1 h, room temperature), followed by the avidin-biotin-peroxidase complex protocol (ABC, Vector, UK), using 3,3′-diaminobenzidine (Merck) as a chromogen. Finally, sections were mounted onto superfrost glass slides coated with gelatin utilizing Eukitt^®^ as mounting medium for visualization.

The immunofluorescence technique was used for the double staining of the ionized calcium-binding adaptor molecule 1 (IBA1) and the interleukin-1β (IL-1β) in SNc, and for the visualization of the TH in CPu. Free-floating sections were rinsed in PB 0.1 M, blocked, and permeabilized in 3% normal goat serum and 0.3% Triton X-100 in 0.1 M PB at room temperature (3 h), followed by incubation in the same solution with the following primary antibodies: rabbit anti-IBA1 (1:600, Wako, Japan, 019-19741), hamster anti-IL-1β (1:50, Santa Cruz Biotech., US, sc-12742), rabbit anti-TH (1:1000, Millipore, US, AB152). Thereafter, sections were rinsed three times in 0.1 M PB, and then incubated with the appropriate AlexaFluor^®^ (1:400, Jackson ImmunoResearch Europe, UK) secondary antibody in 0.1 M PB at room temperature (2 h). Afterward, sections were rinsed and immediately mounted onto superfrost glass slides coated with gelatin by using Mowiol^®^ as the mounting medium. In each of the reaction protocols, the omission of either the primary or secondary antibodies served as negative control and yielded no labelling (data not shown).

#### 2.6.2. Image acquisition and analysis

Images of a single wavelength (14-bit depth) were obtained with the ZEISS Axio Scan.Z1 slide scanner (Zeiss, Germany) connected to either the Hitachi HV-F203SCL (brightfield imaging, 1600 × 1200 pixels, Hitachi Kokusai Electric, Japan) or the Axiocam 506 digital camera (fluorescence imaging, 2752 × 2208 pixels, Zeiss, Germany), and equipped with the LED light source Colibri 2. Brain sections immunostained for TH, IBA1, and IL-1β were captured at 20× magnification (Objective: Plan-Apochromat 20×/0.8 M27) to acquire the whole CPu and the SNc.

Additionally, for colocalization analysis, confocal images of a single wavelength (16-bit depth) were digitalized and captured by using a motorized (Mad City Labs MCL MOTZN) inverted epifluorescence microscope (Zeiss Axio Observer A1; objective: Plan-Apochromat 63×/1,40, OIL M27) equipped with the CREST CARVII spinning disk system and connected to the Prime BSI sCMO S digital camera (4.2 Megapixels; Teledyne photometrics, US).

The ImageJ software (National Institutes of Health, United States) was used to quantify: (1) the density of TH positive fibers in CPu, (2) the mean intensity and the total number of IBA1 positive cells in the SNc, (3) the mean intensity of IL-1β in the SNc, (4) IBA1-IL-1β colocalization in the SNc. For mean intensity quantification, images were background subtracted, then optical density was determined by measuring the mean grey density in fixed regions representing the dorsal CPu and the SNc. Quantification of IBA1 positive cells was carried out by the manual particle counting option of Image J software, while quantitative analysis of co-localization of IBA1 with IL1β was conducted using the ImageJ plugin JACoP (Just Another Co-localisation Plugin) (Bolte and Cordelières, 2006) and calculated as Pearson correlation coefficient (Rr), as previously described (Pinna et al., 2021).

### 2.7. Statistical analysis

Statistical analysis was carried out with GraphPad Prism 8 software (GraphPad Software, Inc., La Jolla, CA), and data were analysed either by unpaired *t*-test (monkeys) or Two-way analysis of variance (ANOVA) followed by Tukey’s post-hoc test (mice), when applicable. Results were considered statistically significant if *p* < 0.05.

## 3. Results

### 3.1. Effect of MPTP-treatment on d-serine and l-serine levels in the rostral putamen of Macaca Mulatta monkeys

**Fig. 1.**
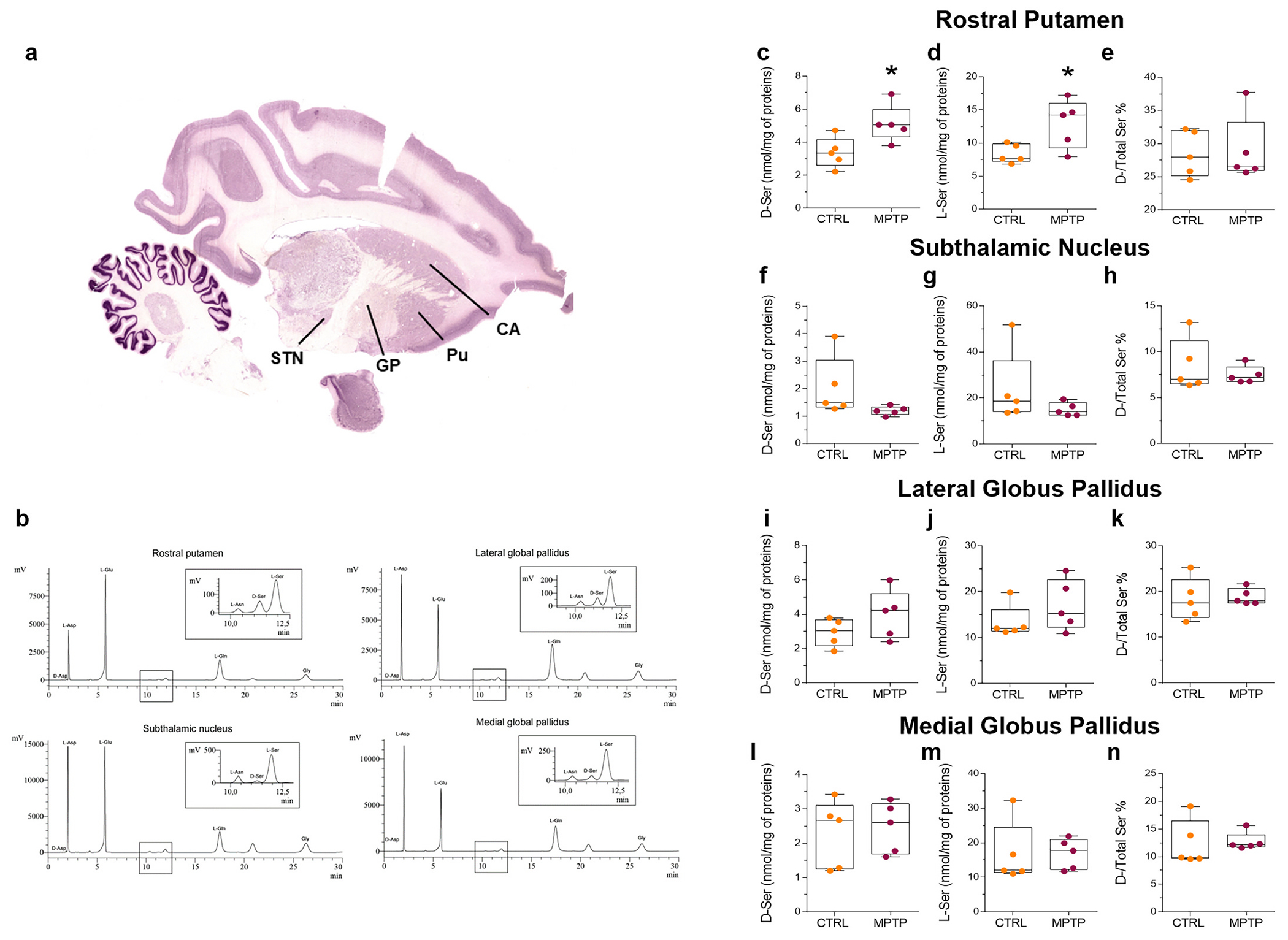
Upregulated d-serine and l-serine levels in the rostral putamen of MPTP-treated monkeys. (**a**) Representative coronal brain section of *Macaca Mulatta* monkeys (Mikula et al., 2007), showing the brain areas analysed in the present study. (**b**) Representative chromatograms. Box plots indicating the concentration of d-serine, l-serine, and the d-/Total serine ratio in the rostral putamen (**c-e**; median [IQR] of nmol/mg of protein; d-serine: CTRL = 3.348 [2.594; 4.181] vs MPTP = 5.064 [4.3; 5.993], *p* = 0.0275; l-serine: CTRL = 7.653 [7.232; 9.87] vs MPTP = 14.24 [9.237; 15.97], *p* = 0.0324; d-/Total serine ratio: CTRL vs MPTP, *p* = 0.8694; unpaired *t*-test), subthalamic nucleus (**f-h**; d-serine: CTRL vs MPTP, *p* = 0.1259; l-serine: CTRL vs MPTP, *p* = 0.2579; d-/Total serine ratio: CTRL vs MPTP, *p* = 0.4697; unpaired t-test), lateral globus pallidus (**i-k**; d-serine: CTRL vs MPTP, *p* = 0.1931; l-serine: CTRL vs MPTP, *p* = 0.2568; d-/Total serine ratio: CTRL vs MPTP, *p* = 0.7949; unpaired t-test) and medial globus pallidus (**l-n**; d-serine: CTRL vs MPTP, *p* = 0.7506; l-serine: CTRL vs MPTP, *p* = 0.9964; d-/Total serine ratio: CTRL vs MPTP, *p* = 0.8771; unpaired t-test) of control and MPTP-treated monkeys (*n* = 5 monkeys/treatment). Box plots indicate the top and bottom quartiles; whiskers refer to the top and bottom 90%. * *p* < 0.05 compared to controls. Abbreviations: CA, caudate; CTRL, control; GP, globus pallidus; MPTP, 1-methyl-4-phenyl-1.2.3.6-tetrahydropyridine; Pu, putamen; STN, subthalamic nucleus. Colour legend: orange. CTRL; red. MPTP. (For interpretation of the references to colour in this figure legend, the reader is referred to the web version of this article.)

First, to determine whether MPTP-induced nigrostriatal degeneration affects the concentration of serine enantiomers, glycine or other d- and l-amino acids involved in NMDAR activation, we performed HPLC analyses in the rPut, STN, lateral and medial part of GP of MPTP-treated monkeys and related controls (Fig. 1, supplemental Fig. 1). We observed increased levels of the endogenous NMDAR co-agonist, d-serine (Fig. 1C; *p* < 0.05), as well as of its metabolic precursor, l-serine (Fig. 1D; p < 0.05), exclusively in the rPut (Fig. 1C,D; p < 0.05) of MPTP-treated monkeys compared to controls. In contrast, we found no differences between parkinsonian monkeys and controls in the d-serine/total serine ratio in the rPut (Fig. 1E; *p* > 0.05). We found no differences in levels of serine enantiomers and the d-serine/total serine ratio in the STN (Fig. 1F–H; p > 0.05), lateral GP (Fig. 1I–K; p > 0.05) and medial GP (Fig. 1L–N; p > 0.05). The variation in the rPut d-serine content is in line with previous data obtained in bilaterally 6-hydroxydopamine (6-OHDA)-lesioned rats (El Arfani et al., 2015) and extends our previous report in the commissural dorsal putamen of MPTP-treated monkeys, where a trend towards increased d-serine and l-serine levels was detected (Nuzzo et al., 2019). Intriguingly, these data are coherent with our report indicating a selective upregulation of serine enantiomers in the *post-mortem* CPu of PD patients (Di Maio et al., 2023). Conversely, we found no differences in the amount of aspartate enantiomers and the d-aspartate/total aspartate ratio in any brain region analysed (Supplemental Fig. S1; *p* > 0.05). No differences were found in l-glutamate, l-glutamine, glycine, and l-asparagine levels in the rPut, STN, lateral and medial GP of MPTP-treated monkeys compared to controls (Supplemental Fig. S1; p > 0.05).

### 3.2. MPTP treatment perturbs GAPDH and ASCT1 protein levels in the rostral putamen of Macaca Mulatta monkeys

**Fig. 2.**
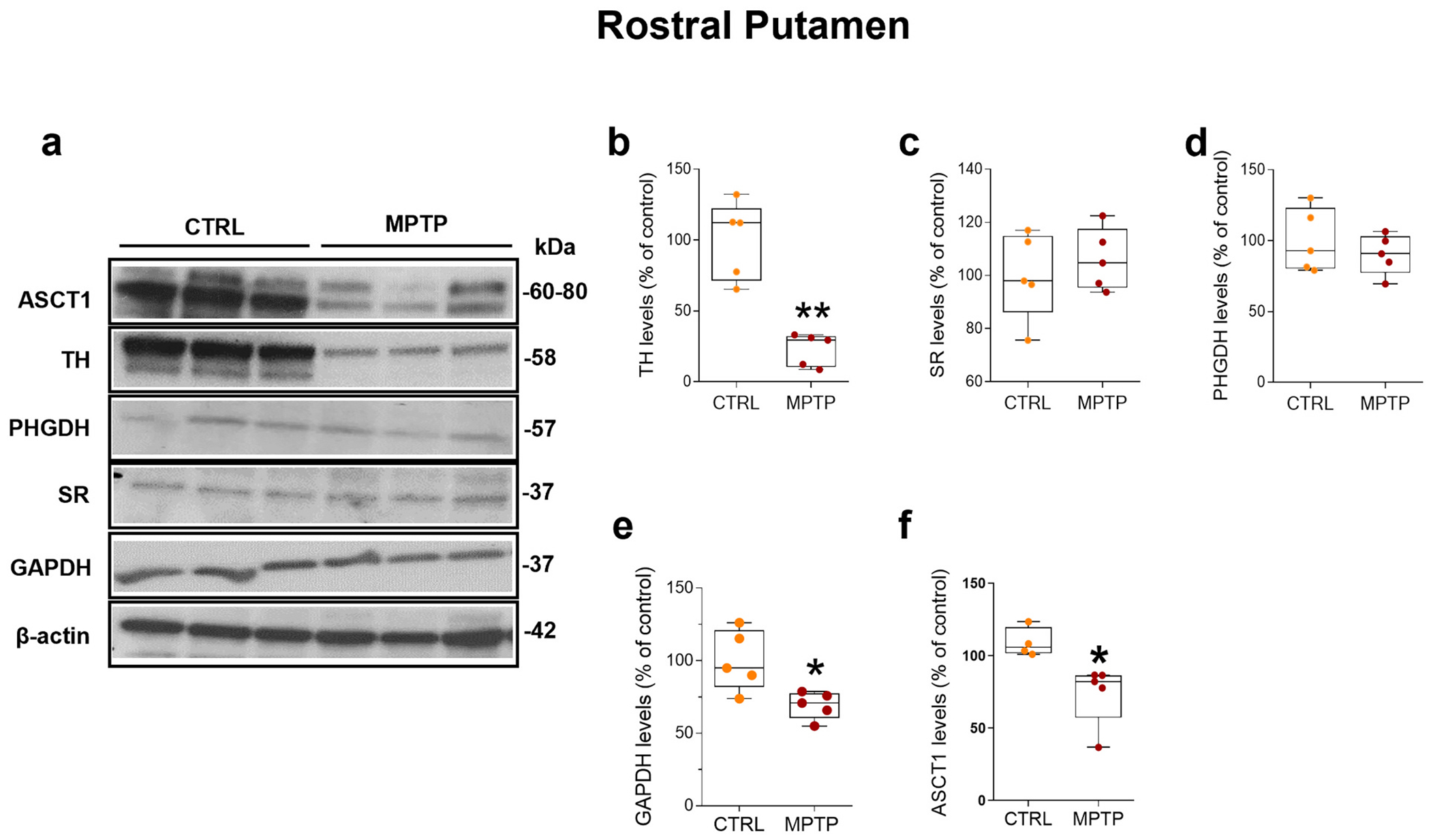
MPTP treatment markedly reduces glyceraldehyde 3-phosphate dehydrogenase and alanine serine cysteine transporter 1 protein levels in the rostral putamen of MPTP-treated monkeys. Representative blots of (**a**) alanine serine cysteine transporter 1 (ASCT1), tyrosine hydroxylase (TH), 3-phosphoglycerate dehydrogenase (PHGDH), serine racemase (SR), glyceraldehyde-3-phosphate dehydrogenase (GAPDH), and β-actin in control (*n* = 3) and MPTP-treated monkeys (n = 3). Box plots indicating protein levels of (**b**) TH (median [IQR] of protein levels; CTRL = 112.2 [71.51; 122.4] vs MPTP = 29.5 [10.52; 32.11], *p* = 0.0004, unpaired *t*-test), (**c**) SR (CTRL vs MPTP, *p* = 0.5151; unpaired t-test), (**d**) PHGDH (CTRL vs MPTP, *p* = 0.4378; unpaired t-test), (**e**) GAPDH (median [IQR] of protein levels; CTRL = 94.85 [81.88; 120.7] vs MPTP = 70.86 [60.39; 77.29], *p* = 0.0317, unpaired t-test), and (**f**) ASCT1 (median [IQR] of protein levels; CTRL = 105.8 [101.6; 119.7] vs MPTP = 82.24 [57.21; 86.54], *p* = 0.0193, unpaired t-test) identified by Western blot in the rostral putamen of control and MPTP-treated monkeys (*n* = 5 monkeys/treatment). Box plots indicate the top and bottom quartiles; whiskers refer to the top and bottom 90%. TH, SR, PHGDH, GAPDH, and ASCT1 variations are expressed as a percentage of the control group. β-actin was used as an internal control to normalize the variation in protein loading and transfer. * *p* < 0.05, ** *p* < 0.01 compared to controls. Abbreviations: CTRL, control; MPTP, 1-methyl-4-phenyl-1.2.3.6-tetrahydropyridine. Colour legend: orange. CTRL; red. MPTP. (For interpretation of the references to colour in this figure legend, the reader is referred to the web version of this article.)

Next, we measured the extent of MPTP-induced dopaminergic neurodegeneration in the rPut of controls and MPTP-treated monkeys. WB analysis showed that MPTP treatment produced a decrease of about 75% in the rPut protein content of tyrosine hydroxylase (TH), a specific marker of dopaminergic neurons (Fig. 2A,B; *p* < 0.01).

We then examined the rPut level of serine racemase (SR) and d-amino acid oxidase (DAAO), the two major enzymes involved in the regulation of d-serine metabolism (Canu et al., 2014; Sasabe and Suzuki, 2019; Seckler and Lewis, 2020; Wolosker et al., 2016), as well as of 3-phosphoglycerate dehydrogenase (PHGDH), the astrocytic rate limiting enzyme regulating the de novo synthesis of l-serine (Grant, 2018; Murtas et al., 2021; Murtas et al., 2020). WB data showed comparable protein levels of both SR (Fig. 2A,C; *p* > 0.05) and PHGDH (Fig. 2A,D; p > 0.05) between control and MPTP-treated monkeys. On the other hand, in accordance with prior investigations in the forebrain of rodents, monkeys and humans (Di Maio et al., 2023; Gonda et al., 2022; Nuzzo et al., 2019; Suzuki et al., 2017), we found that DAAO levels were below the limit of WB detection.

Thereafter, we measured the protein levels of glyceraldehyde-3-phosphate dehydrogenase (GAPDH), a glycolytic enzyme that converts glyceraldehyde 3-phosphate to glycerate-1,3-biphosphate, that was previously found to regulate SR activity (Suzuki et al., 2015). We found a significant reduction in the rPut protein levels of GAPDH in MPTP-treated monkeys compared to controls (Fig. 2A,E; *p* < 0.05). In the same brain region, we measured the concentration of ASCT1, a transmembrane protein involved in the shuttle of d-serine and l-serine between neurons and astrocytes (Kaplan et al., 2018). We found that MPTP treatment significantly decreased the protein levels of ASCT1 in the rPut (Fig. 2A,F; p < 0.05).

### 3.3. Effects of sub-chronic MPTPp treatment and oral d-serine supplementation on the extent of dopaminergic nigrostriatal degeneration in mice

**Fig. 3.**
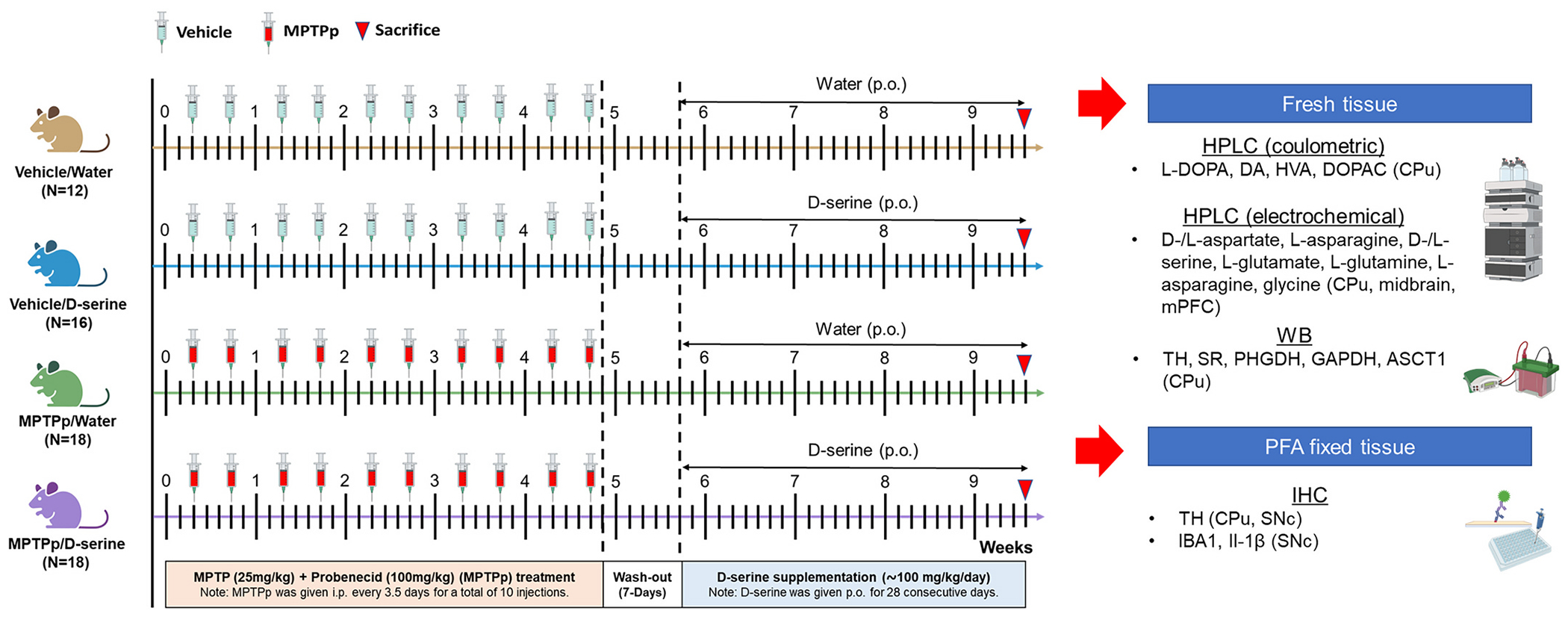
Experimental plan: oral d-serine supplementation in sub-chronically MPTPp-treated mice. Adult 12-weeks old C57BL/6 J mice received an intraperitoneal (i. p.) injection of either saline (vehicle) or MPTP (25 mg/kg) plus probenecid (100 mg/kg; 30 mins prior to MPTP administration) (MPTPp), twice a week for 5-consecutive weeks. Thereafter, mice were subjected to a drug wash-out period of 7 days, followed by either oral d-serine supplementation (~100 mg/kg/day) in the drinking water or standard drinking water for one month (28 days). Lastly, mice were sacrificed either by transcardial perfusion, for further immunohistochemical analyses, or by decapitation, for HPLC and Western blot analyses. Abbreviations: ASCT1, alanine serine cysteine transporter 1; CPu: caudate putamen; DA, dopamine; DOPAC, 3,4-Dihydroxyphenylacetic acid; GAPDH, glyceraldehyde-3-phosphate dehydrogenase; HPLC, High-performance liquid chromatography; HVA, homovanillic acid; IBA1, ionized calcium-binding adapter molecule 1; Il-1β, interleukin-1β; L-DOPA, levodopa; mPFC, medial prefrontal cortex; MPTP, 1-methyl-4-phenyl-1,2,3,6-tetrahydropyridine; MPTPp, MPTP plus probenecid; p.o., oral; PHGDH, 3-phosphoglycerate dehydrogenase; SNc, substantia nigra pars compacta; SR, serine racemase; TH, tyrosine hydroxylase; WB, western blot.

**Fig. 4.**
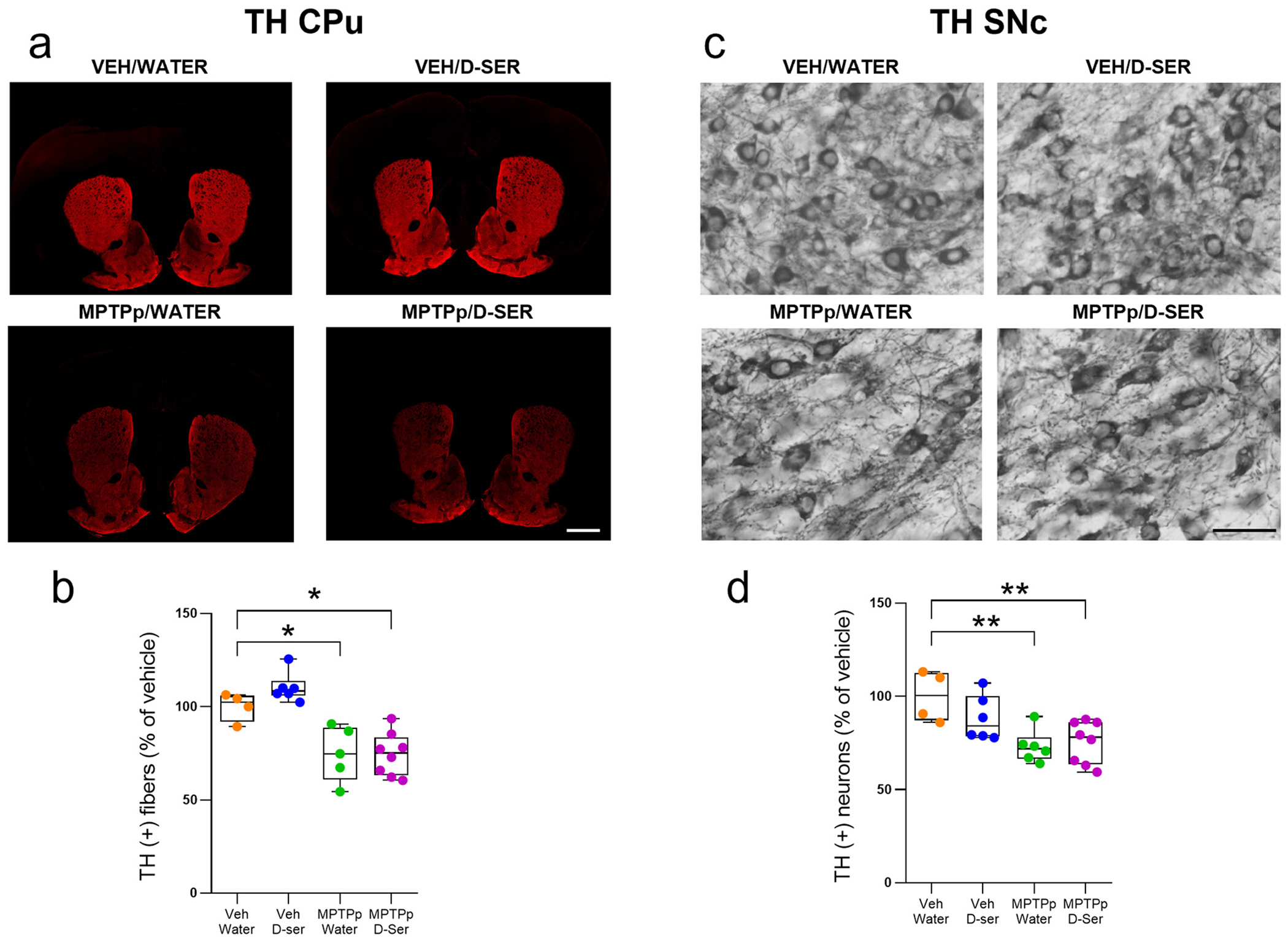
Oral d-serine supplementation does not affect the degeneration of nigrostriatal dopaminergic neurons and terminals in MPTPp-treated mice. (**a**) Representative images of the caudate putamen (CPu) immunostained for tyrosine hydroxylase (TH). (**b**) Box plots indicating the mean intensity of TH-(+) fibers in the dorsal CPu (median [IQR] of TH (+) fibers; Veh/Water = 102.2 [91.99; 105.8] vs MPTPp/Water = 74.72 [60.88; 88.78], *p* = 0.0166; Veh/Water = 102.2 [91.99; 105.8] vs MPTPp/d-serine = 75.10 [63.19; 83.46], *p* = 0.0080; Two-way ANOVA followed by Tukey’s post hoc test [MPTPp effect: F_(1,18)_ = 38.29. *p* < 0.0001]). (**c**) Representative images of the substantia nigra pars compacta (SNc) immunostained for TH. (**d**) Box plots indicating the total number of TH-(+) neurons in the SNc (median [IQR] of TH (+) neurons; Veh/Water = 100.4 [87.20; 112.4] vs MPTPp/Water = 71.96 [66.33; 77.98], *p* = 0.0038; Veh/Water = 100.4 [87.20; 112.4] vs MPTPp/d-serine = 78.10 [63.64; 86.04], *p* = 0.0058; Two-way ANOVA followed by Tukey’s post hoc test [MPTPp effect: F_(1,20)_ = 17.40. *p* = 0.0005]). Box plots indicate the top and bottom quartiles; whiskers refer to the top and bottom 90%. TH variation is expressed as a percentage of the control group. * *p* < 0.05, ** *p* < 0.01 compared to Veh/Water. Scale bar = 1000 μm (CPu); 50 μm (SNc). Abbreviations: D-ser, d-serine; MPTP, 1-methyl-4-phenyl-1,2,3,6-tetrahydropyridine; MPTPp, MPTP+probenecid; Veh, vehicle. Colour legend: orange. Veh/Water; blue. Veh/D-ser; green. MPTPp/Water; violet. MPTPp/D-ser. (For interpretation of the references to colour in this figure legend, the reader is referred to the web version of this article.)

To investigate the consequence of a mild dopaminergic denervation on striatal serine enantiomers levels as well as on ASCT1 and GAPDH expression, and to assess the potential modulatory effects of d-serine administration (~100 mg/kg/day), here we used a sub-chronic MPTPp treatment that elicited a minor but significant loss of TH immunoreactivity throughout the CPu (Figs. 3,4). We found that sub-chronic administration of MPTPp decreased striatal TH+ fiber density (Fig. 4A,B; *p* < 0.05) and nigral TH+ neurons (Fig. 4C–D; *p* < 0.01) of about 25% and 27%, respectively, compared to control vehicle/water-treated mice. Moreover, one month of oral d-serine supplementation did not affect TH immunoreactivity in the CPu and SNc, regardless of MPTPp treatment (Fig. 4A–D; *p* > 0.05), demonstrating that exogenous d-serine was safe and did not affect the dopaminergic nigrostriatal neurotoxic effects of MPTPp (Fig. 4A–D).

### 3.4. Effects of sub-chronic MPTPp treatment and oral d-serine supplementation on striatal dopamine metabolism in mice

**Fig. 5.**
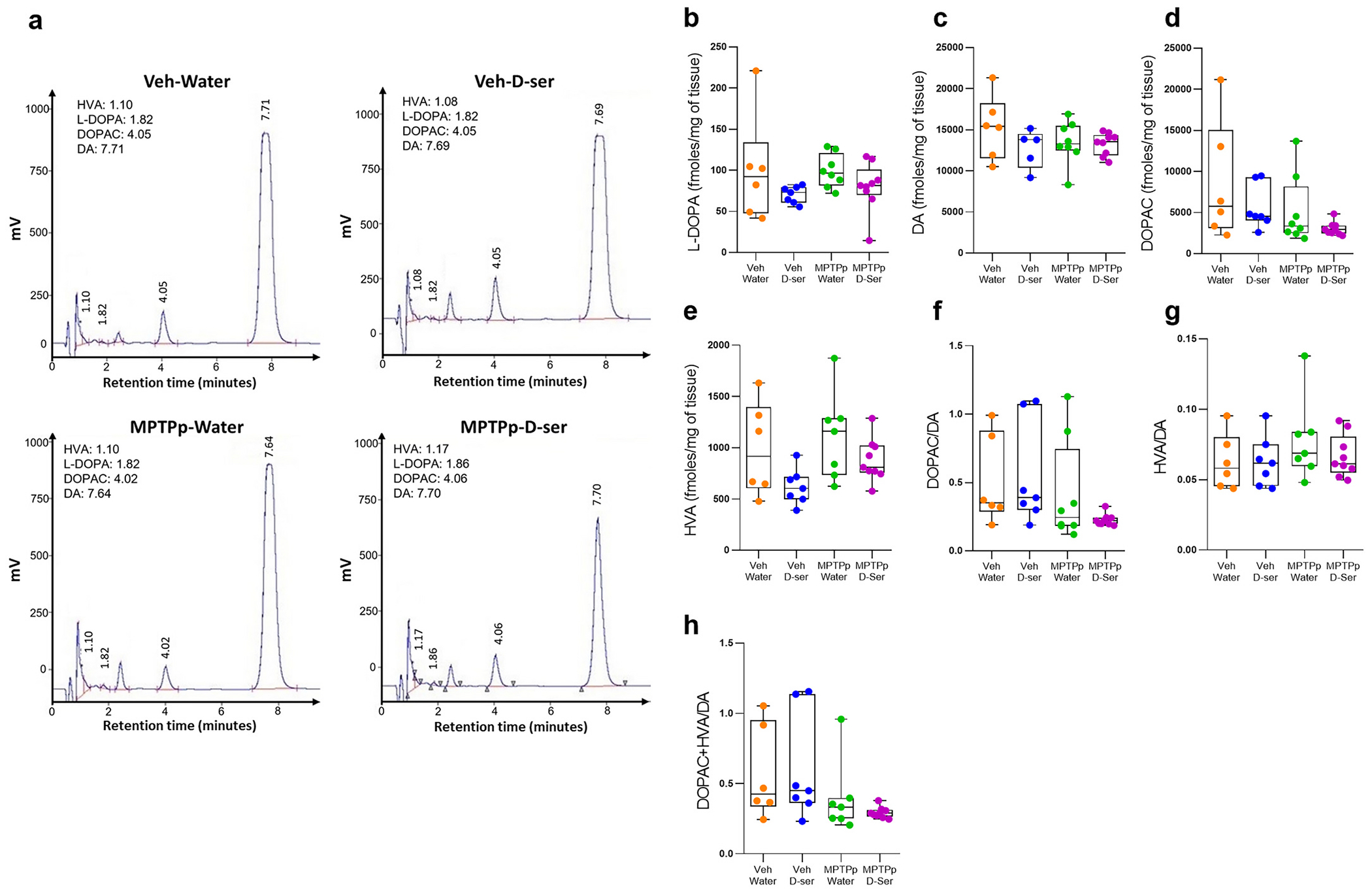
Oral d-serine supplementation does not affect striatal dopamine metabolism in vehicle- and MPTPp-treated mice. (**a**) Representative chromatograms for homovanillic acid (HVA), levodopa (L-DOPA), 3,4-dihydroxyphenylacetic acid (DOPAC), and dopamine (DA) from the caudate putamen of Veh/Water, Veh/D-ser, MPTPp/Water, and MPTPp/D-ser-treated mice (*n* = 6-9 mice/treatment). The numbers above peaks indicate the retention time. (**b-h**) Box plots indicating striatal concentration of L-DOPA (**b,** p > 0.05), DA (**c,** p > 0.05), DOPAC (**d,** p > 0.05), HVA (**e,** p > 0.05), and the ratio of DOPAC/DA (**f,** p > 0.05), HVA/DA (**g,** p > 0.05), DOPAC+HVA/DA (**h,** p > 0.05) (Two-way ANOVA followed by Tukey’s post hoc test, p > 0.05). Box plots indicate the top and bottom quartiles; whiskers refer to top and bottom 90%. *N* = 6-9 mice/treatment. Abbreviations: D-ser, d-serine; MPTP, 1-methyl-4-phenyl-1,2,3,6-tetrahydropyridine; MPTPp, MPTP+probenecid; Veh, vehicle. Colour legend: orange, Veh/Water; blue, Veh/D-ser; green, MPTPp/Water; violet, MPTPp/D-ser. (For interpretation of the references to colour in this figure legend, the reader is referred to the web version of this article.)

Next, to evaluate the effect of sub-chronic MPTPp treatment and oral d-serine supplementation on the metabolism of striatal DA in treated mice, we analysed striatal levels of levodopa (L-DOPA), DA, and its metabolites, 3,4-dihydroxyphenylacetic acid (DOPAC) and homovanillic acid (HVA). HPLC analysis revealed that sub-chronic MPTPp treatment did not significantly alter the levels of L-DOPA, DA, and its metabolites compared to control vehicle/water-treated mice (Fig. 5A–E; *p* > 0.05). Similarly, the ratios of DOPAC/DA, HVA/DA, and DOP-AC+HVA/DA were unaffected (Fig. 5F–H; *p* > 0.05). Regardless of the MPTPp treatment, oral d-serine supplementation did not affect the striatal dopaminergic metabolism, as indicated by unaltered levels of L-DOPA, DA, DOPAC, and HVA, or the ratios of DOPAC/DA, HVA/DA, and DOPAC+HVA/DA compared to control vehicle/water-treated mice (Fig. 5A–H; p > 0.05).

### 3.5. Oral d-serine supplementation elevates the d-serine/total serine ratio in the caudate putamen, midbrain, and medial prefrontal cortex of treated mice

**Fig. 6.**
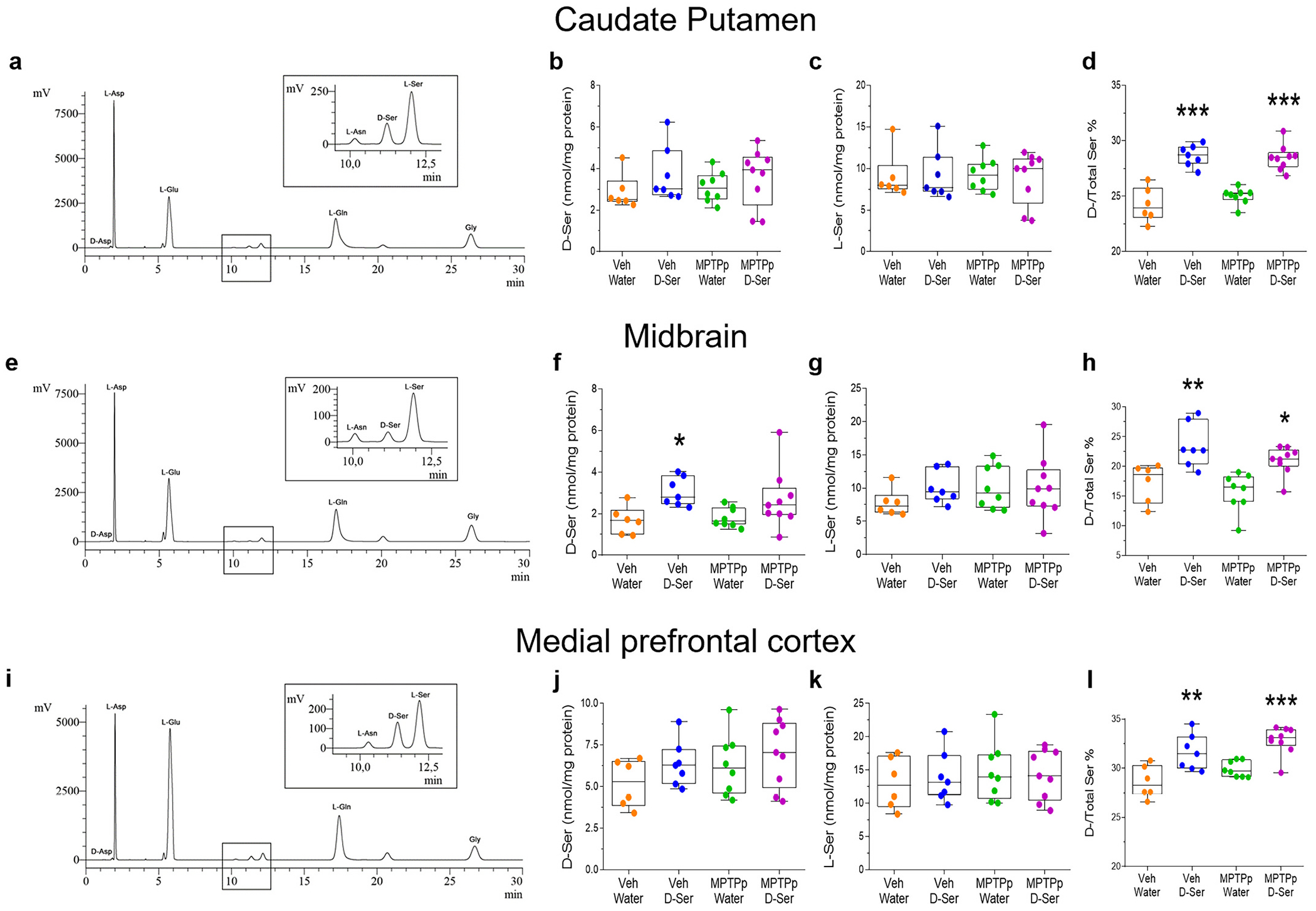
Oral d-serine supplementation increases the d-/total serine ratio in the caudate putamen, midbrain, and medial prefrontal cortex of vehicle- and MPTPp-treated mice. **(a)** Representative chromatogram and **(b-d)** box plots indicating the concentration of d-serine (**b;** *p* > 0.05), l-serine (**c;** p > 0.05), and the d-/Total serine ratio in the caudate putamen (CPu) (**d**; median [IQR] of d-/Total serine ratio; Veh/Water = 23.92 [23.03; 25.77] vs Veh/D-Ser = 28.71 [27.93; 29.46], *p* < 0.0001; Veh/Water = 23.92 [23.03; 25.77] vs MPTPp/D-Ser = 28.49 [27.63; 28.96], *p* < 0.0001; Two-way ANOVA followed by Tukey’s post hoc test [d-serine effect: F(_1,26_) = 92.94, p < 0.0001]). (**e**) Representative chromatogram and (**f-h**) box plots indicating the concentration of d-serine (**f**; median [IQR] of nmol/mg protein; Veh/Water = 1.658 [0.9904; 2.176] vs Veh/D-Ser = 2.79 [2.446; 3.832], *p* = 0.0351; Two-way ANOVA followed by Tukey’s post hoc test [d-serine effect: F_(1,26)_ = 10.41, *p* = 0.0034]), l-serine (**g**; p > 0.05), and the d-/Total serine ratio in the midbrain (**h**; median [IQR] of d-/Total serine ratio: Veh/Water = 18.59 [13.73; 19.76] vs Veh/D-Ser = 22.7 [20.36; 27.96], *p* = 0.0033; Veh/Water = 18.59 [13.73; 19.76] vs MPTPp/D-Ser = 21.26 [20; 22.79], *p* = 0.0429; Two-way ANOVA followed by Tukey’s post hoc test [d-serine effect: F_(1,26)_ = 25.44, p < 0.0001]). (**i**) Representative chromatogram and (**j-l**) box plots indicating the concentration of d-serine (**j**; p > 0.05), l-serine (**k**; p > 0.05), and the d-/Total serine ratio in the prefrontal cortex (**l;** median [IQR] of d-/Total serine ratio: Veh/Water = 28.27 [27.33; 30.31] vs Veh/D-Ser = 31.46 [29.97; 33.19], *p* = 0.0023; Veh/Water = 28.27 [27.33; 30.31] vs MPTPp/D-Ser = 33.09 [32.28; 33.94], p < 0.0001; Two-way ANOVA followed by Tukey’s post hoc test [MPTPp effect: F_(1,26)_ = 5.543, *p* = 0.0264; d-serine effect: F_(1,26)_ = 30.98, p < 0.0001]). Box plots indicate the top and bottom quartiles; whiskers refer to the top and bottom 90%. *N* = 6-9 mice/treatment. * *p* < 0.05, ** *p* < 0.01, and *** *p* < 0.001 compared to Veh/Water. Abbreviations: D-ser, d-serine; MPTP, 1-methyl-4-phenyl-1,2,3,6-tetrahydropyridine; MPTPp, MPTP+probenecid; Veh, vehicle. Colour legend: orange, Veh/Water; blue, Veh/D-ser; green, MPTPp/Water; violet, MPTPp/D-ser. (For interpretation of the references to colour in this figure legend, the reader is referred to the web version of this article.)

Next, we evaluated whether sub-chronic MPTPp treatment and oral d-serine supplementation affect the concentration of serine enantiomers (Fig. 6) and other NMDAR-related amino acids (supplemental Fig. 2) in total homogenates of CPu, midbrain, and medial prefrontal cortex (mPFC) of treated mice.

In contrast to what we found in the rPut of MPTP-treated monkeys, HPLC results highlighted that sub-chronic MPTPp treatment did not alter the levels of serine enantiomers in the CPu of treated mice compared to vehicle/water-treated controls (Fig. 6A–D; *p* > 0.05). Similarly, no modifications in either serine enantiomers levels or their ratio was detected in the midbrain and mPFC of MPTPp-treated mice (Fig. 6E–L; *p* > 0.05).

We found that oral d-serine supplementation significantly elevated the d-serine/total serine ratio in all brain regions analysed, regardless of the MPTPp treatment, compared to control vehicle/water-treated mice (Fig. 6D,H,L; *p* < 0.05). We detected increased d-serine levels only in the midbrain of mice receiving vehicle/d-serine treatment as compared to vehicle/water (Fig. 6F; p < 0.05), and no increase of l-serine in any brain regions analysed (Fig. 6C,G,K; *p* > 0.05).

In addition, our results revealed that, in the CPu, midbrain, and mPFc, neither MPTPp treatment nor oral d-serine supplementation altered the concentration of other NMDAR-related amino acids, including d- and l-aspartate, l-glutamate, l-glutamine, glycine, and l-asparagine (Supplemental Fig. 2).

### 3.6. Unchanged striatal serine enantiomers levels and GAPDH expression in mice subjected to sub-chronic MPTPp treatment and oral d-serine supplementation

**Fig. 7.**
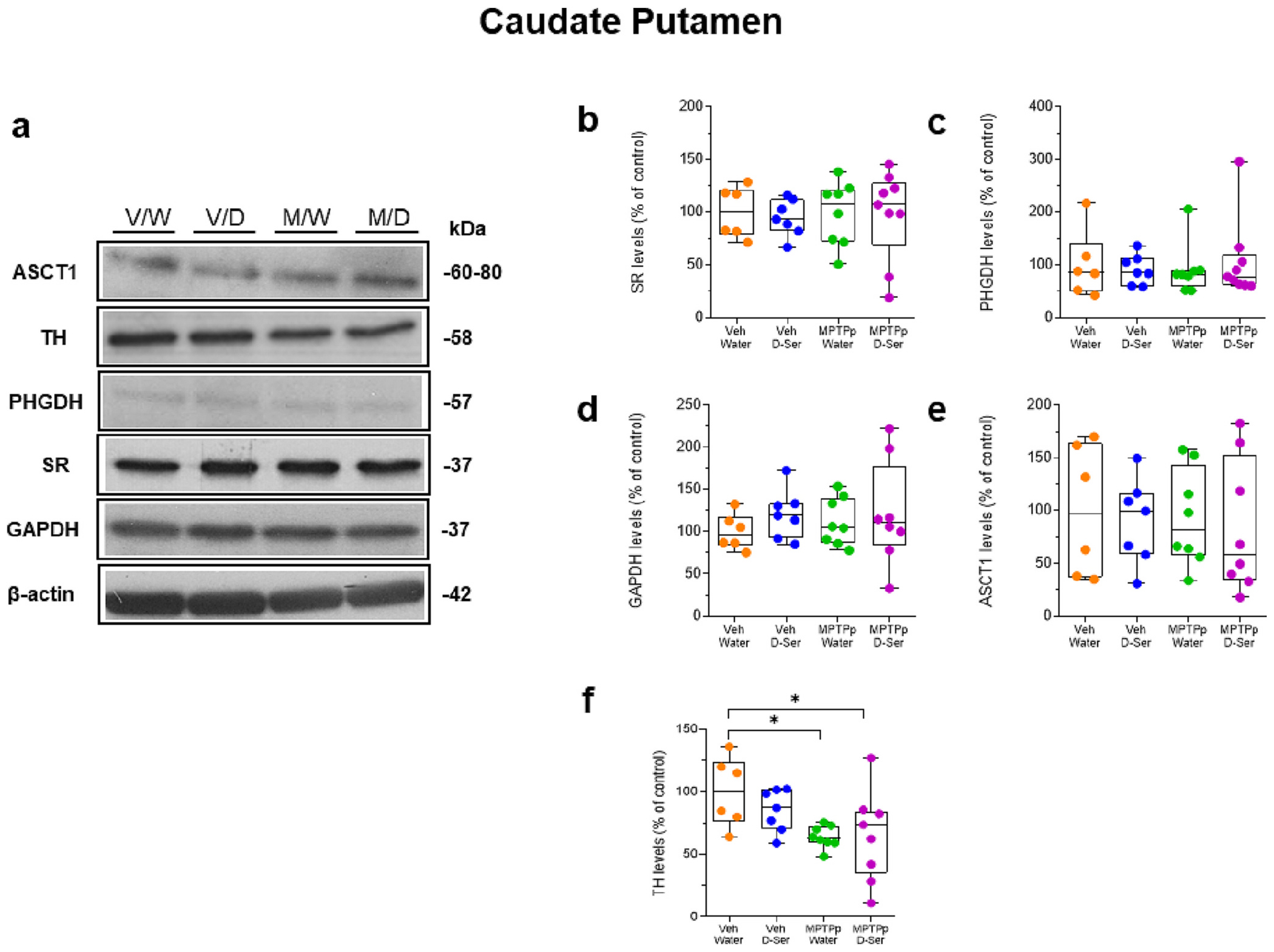
Oral d-serine supplementation did not alter the levels of serine racemase, 3-phosphoglycerate dehydrogenase, glyceraldehyde 3-phosphate dehydrogenase and alanine serine cysteine transporter 1 protein in the caudate putamen of vehicle- and MPTPp-treated mice. Representative blots of **(a)** alanine serine cysteine transporter 1 (ASCT1), tyrosine hydroxylase (TH), 3-phosphoglycerate dehydrogenase (PHGDH), serine racemase (SR), glyceraldehyde-3-phosphate dehydrogenase (GAPDH), and β-actin. Box plots indicating protein levels of SR (**b**; p > 0.05), PHGDH (**c**; p > 0.05), GAPDH (**d**; p > 0.05), ASCT1 (**e**; p > 0.05), and TH (**f;** median [IQR] of TH protein levels; Veh/Water = 100 [75.95; 124] vs MPTPp/Water = 62.74 [59.45; 72.33], *p* = 0.0306; Veh/Water = 100 [75.95; 124] vs MPTPp/D-Ser = 73.49 [35.16; 84.06], *p* = 0.0378; Two-way ANOVA followed by Tukey’s post hoc test [MPTPp effect: F_(1,26)_ = 9.096, *p* = 0.0057]). Box plots indicate the top and bottom quartiles; whiskers refer to the top and bottom 90%. SR, PHGDH, GAPDH, ASCT1, and TH variations are expressed as a percentage of the control group. β-actin was used as an internal control to normalize the variation in protein loading and transfer. *N* = 6-9 mice/treatment. * p < 0.05 compared to Veh/Water. Abbreviations: ASCT1, alanine serine cysteine transporter 1; CTRL, control; GAPDH, glyceraldehyde-3-phosphate dehydrogenase; D-ser, d-serine; MPTP, 1-methyl-4-phenyl-1.2.3.6-tetrahydropyridine; MPTPp, MPTP+probenecid PHGDH, 3-phosphoglycerate dehydrogenase; SR, serine racemase; TH, tyrosine hydroxylase; Veh, vehicle. Colour legend: orange, Veh/Water; blue, Veh/D-ser; green, MPTPp/Water; violet, MPTPp/D-ser. (For interpretation of the references to colour in this figure legend, the reader is referred to the web version of this article.)

Based on the higher d-serine/total serine ratio found in the CPu of vehicle- and MPTPp-treated mice receiving oral d-serine supplementation, we then investigated whether exogenous d-serine might promote compensatory mechanisms affecting the striatal expression of SR and PHGDH. WB analyses indicated that neither sub-chronic MPTPp treatment, oral d-serine supplementation, nor their combination affected striatal protein levels of SR and PHGDH compared to vehicle/water treatment (Fig. 7A–C; *p* > 0.05).

As MPTP treatment in monkeys markedly reduced the putaminal protein levels of the glycolytic enzyme GAPDH and of the astrocytic serine transporter ASCT1, we then asked whether similar alterations might be detected in the CPu of mice receiving sub-chronic MPTPp treatment. Interestingly, we found that sub-chronic MPTPp treatment did not affect striatal protein levels of GAPDH (Fig. 7A,D; *p* > 0.05) and ASCT1 (Fig. 7A,E; p > 0.05) compared to vehicle treatment. Similar results were observed in mice receiving oral d-serine supplementation, without MPTPp treatment (Fig. 7A,D,E; p > 0.05). Consistent with immunohistochemical results indicating a mild degeneration of nigrostriatal dopaminergic fibers, both MPTPp/Water and MPTP/d-serine-treated mice showed a ~30% reduction in the striatal protein levels of TH compared to control vehicle/water-treated mice (Fig. 7A,F; *p* < 0.05).

### 3.7. Oral d-serine supplementation does not activate microglial cells in the substantia nigra pars compacta of vehicle- and MPTPp-treated mice

**Fig. 8.**
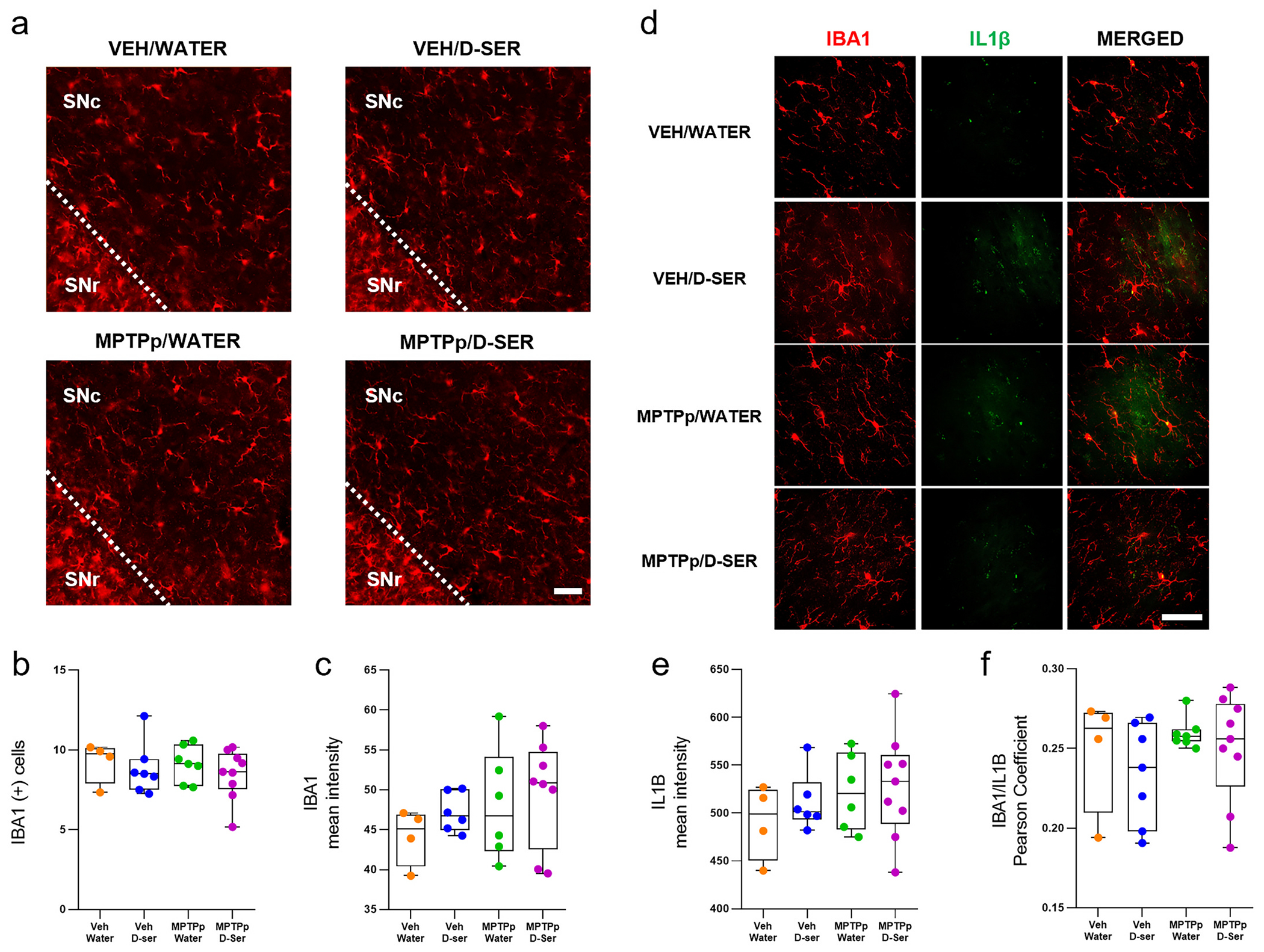
Oral d-serine supplementation does not promote microgliosis in the substantia nigra pars compacta of vehicle- and MPTPp-treated mice. (**a**) Representative images of the substantia nigra pars compacta (SNc) immunostained for the ionized calcium binding adaptor molecule 1 (IBA1) in Veh/Water, Veh/D-ser, MPTPp/Water, and MPTPp/D-ser treated-mice (*n* = 4-9 mice/treatment). (**b-c**) Box plots indicating the total number (**b,** p > 0.05) and the mean intensity (**c**, p > 0.05) of IBA1 (+) cells in the SNc (Two-way ANOVA followed by Tukey’s post hoc test, p > 0.05). Box plots indicate the top and bottom quartiles; whiskers refer to top and bottom 90%. (**d**) Representative confocal images of the SNc immunostained for IBA1 (red, left), interleukin-1β (IL-1β) (green, middle), and IBA1+ IL-1β (yellow, right) in Veh/Water, Veh/D-ser, MPTPp/Water, and MPTPp/D-ser-treated mice (n = 4-9 mice/treatment). (**e-f**) Box plots indicating the mean intensity of IL-1β (**e,** p > 0.05) and the Pearson correlation coefficient for IBA1/IL-1β (**f**, p > 0.05) in the SNc (Two-way ANOVA followed by Tukey’s post hoc test, p > 0.05). Box plots indicate the top and bottom quartiles; whiskers refer to top and bottom 90%. Scale bar = 50 μm. Abbreviations: D-ser, D-serine; MPTP, 1-methyl-4-phenyl-1,2,3,6-tetrahydropyridine; MPTPp, MPTP+probenecid; Veh, Vehicle. Colour legend: orange, Veh/Water; blue, Veh/D-ser; green, MPTPp/Water; violet, MPTPp/D-ser. (For interpretation of the references to colour in this figure legend, the reader is referred to the web version of this article.)

Elevated release of d-serine from reactive glial cells has been hypothesized to promote synaptic dysfunction and excitotoxicity in pathological conditions (Beltrán-Castillo et al., 2018; Coyle et al., 2020; Perez et al., 2017; Tapanes et al., 2022). However, to our knowledge, no study has assessed whether exogenous d-serine supplementation may affect the reactivity of microglial cells in the nigrostriatal pathway of a PD mouse model. To this end, we evaluated the immunoreactivity of the ionized calcium-binding adaptor molecule 1 (IBA1), a specific marker of microglial cells and circulating macrophages, and the pro-inflammatory interleukin-1β (IL-1β) in the SNc (Fig. 8). We found that administration of MPTPp did not alter the number and the mean intensity of IBA1+ cells compared to control vehicle/water-treated mice in the SNc (Fig. 8A–C; *p* > 0.05). Similarly, the mean intensity of IL-1β, and IBA1/IL-1β colocalization, did not differ between vehicle- and MPTPp-treated mice (Fig. 8D–F; *p* > 0.05). These results corroborate previous reports indicating that MPTPp-induced microglial activation reaches a peak within the 24 h following treatment cessation, and then progressively returns to normal (Belloli et al., 2017; Schintu et al., 2009). Importantly, our data indicate the lack of detectable pro-inflammatory events associated with exogenous d-serine administration. Indeed, irrespective of MPTPp treatment, mice receiving one month of oral d-serine supplementation (100 mg/kg/day) showed no changes in the immunoreactivity for IBA1, IL-1β, and IBA1/IL-1β colocalization in the SNc as compared to control vehicle/water-treated mice (Fig. 8A–F; *p* > 0.05).

## 4. Discussion

In the present study, we evaluated the consequences of MPTP-induced dopaminergic midbrain degeneration on the cerebral concentration of d-serine and l-serine in two well-established animal models of PD, i.e., the MPTP-treated macaque and the MPTPp-treated mouse.

We found that MPTP treatment in monkeys induced a severe nigrostriatal dopaminergic denervation coupled with a robust elevation of both d-serine and l-serine in the rPut compared to controls. Importantly, the elevation of serine enantiomers was brain region-specific, as the levels of both amino acids were comparable to controls in other basal ganglia areas including the STN, and the medial/lateral GP. Surprisingly, WB analyses revealed comparable protein levels of SR and PHGDH, the two key enzymes regulating the *de novo* biosynthesis of d-serine (Canu et al., 2014; Coyle et al., 2020; Wolosker et al., 2016) and l-serine (Grant, 2018; Murtas et al., 2021; Murtas et al., 2020), respectively, in the rPut of MPTP-treated and control monkeys. Although further investigations on larger cohorts of monkeys and complementary methodological approaches are warranted, these findings resemble those obtained in a recent clinical study by our group (Di Maio et al., 2023), in which the levels of both serine enantiomers were significantly increased in the *post-mortem* CPu and in the CSF of *de novo* living PD patients, irrespective of the Braak Lewy Body stage (3-4 and 6), compared to control subjects. Considering that MPTP-treated monkeys had never received any treatment and their brains were rapidly frozen after death, these results suggest that the increased levels of serine enantiomers observed in the *post-mortem* CPu of individuals with PD are unlikely to be caused by PD medications or *post-mortem* interval (PMI). Rather, we presume that the detected variations in both d-serine and l-serine levels may reflect a complex alteration in the neurometabolism, and in particular in the aerobic glycolysis. In support of this assumption, widespread abnormalities in cerebral glucose metabolism, as well as impairment in glycolysis and neuronal energy demand have been documented in the PD brain (Barinova et al., 2018; Dai et al., 2023; Saxena, 2012). In addition, previous studies have demonstrated that, in astrocytes, the glycolytic flux significantly affects the synthesis of d-serine and l-serine through distinct mechanisms (Le Douce et al., 2020; Suzuki et al., 2015). Specifically, in the 3xTg mouse model of AD, it has been shown that, in the early stages of the disease, glycolysis impairment significantly compromises the astrocytic synthesis of l-serine, resulting in a subsequent decrease in d-serine content (Le Douce et al., 2020). On the other hand, an in vitro study in primary cultured astrocytes revealed that GAPDH directly interacts with and inhibits SR, thereby suppressing the astrocytic production of d-serine (Suzuki et al., 2015). Thus, these findings suggest that the increased rPut d-serine levels observed in parkinsonian monkeys might depend, at least in part, on the altered expression of the glycolytic enzyme GAPDH.

As a potential indication of astrocytes involvement in the present neurochemical observations, neuroanatomical examinations on *post-mortem* CPu samples from PD patients and various animal models of PD, such as reserpine-treated mice, 6-OHDA-lesioned rats, and MPTP-treated monkeys, demonstrated that DA depletion leads to significant striatal astrogliosis and increased cell contacts between astrocytes and neurons at glutamatergic synapses (Charron et al., 2014). Given that astrocytes are the sole cellular population responsible for l-serine synthesis in the brain (Furuya et al., 2000; Rabattoni et al., 2023; Yamasaki et al., 2001), and that alterations in glucose metabolism have been recently reported in human induced pluripotent stem cells (iPSC)-derived astrocytes from familiar PD patients (Sonninen et al., 2020), we infer that the changes in rPut l-serine concentration in MPTP-treated monkeys may reflect the presence of a greater number of activated astrocytes (Charron et al., 2014). In the same way, striatal astrogliosis may also contribute to the elevation of putaminal d-serine levels as highlighted by prior studies indicating a switch in d-serine production from neurons to astrocytes and microglial cells under neuropathological circumstances (Coyle et al., 2020; Perez et al., 2017; Tapanes et al., 2022; Wolosker et al., 2016).

As a further indication of the emerging role of astrocytes in PD pathophysiology (Booth et al., 2017; Wang et al., 2021) and in agreement with *post-mortem* findings in the CPu of PD patients (Di Maio et al., 2023), we found lower ASCT1 levels in the rPut of MPTP-treated monkeys compared to controls. Hence, we argue that the reduction in ASCT1 levels could represent a secondary event resulting from the excessive utilization of the transporter in response to abnormally higher putami- nal levels of its ligands.

Interestingly, a previous study conducted in ASCT-1 knock-out mice demonstrated the specific expression of this transporter in astrocytes (Kaplan et al., 2018), along with its pivotal function in regulating the extracellular levels of d-serine through the trafficking of serine enantiomers between astrocytes and neurons (Kaplan et al., 2018; Krishnan and Billups, 2023). Therefore, we cannot rule out that downregulation in putaminal ASCT-1 protein levels in parkinsonian monkeys might significantly affect striatal NMDAR occupancy by altering the synaptic concentration of this atypical NMDAR co-agonist. However, further studies are required to corroborate this hypothesis and to determine how the dysregulation in d-serine levels, found in rPut homogenates, could impact the activation of striatal NMDAR receptors at both pre- and post-synaptic compartments. Similarly, it remains to be determined whether the mechanisms underlying the altered expression of GAPDH and ASCT1 reflect an adaptive response stemming from a broad astrocytic dysfunction secondary to dopamine denervation.

Irrespective of the still unknown underlying mechanisms, previous (Nuzzo et al., 2019) and current results highlight that d-serine metabolism in the basal ganglia of PD monkeys undergoes different region-specific variations compared to the brain of control monkeys. Indeed, in MPTP-lesioned monkeys, there was a tendency towards increased d-serine concentration in the post-commissural dorsal putamen (Nuzzo et al., 2019), accompanied by a significant upregulation of this neuroactive molecule in the rPut. Conversely, no variations in the levels of d-serine were detected in the STN, lateral and medial GP, while a marked reduction of this NMDAR co-agonist, along with a pronounced enhancement of DAAO mRNA and protein expression, was found in the substantia nigra (Nuzzo et al., 2019) of MPTP-lesioned monkeys.

Although further studies are warranted to address this key point, we hypothesized that the opposite variations in d-serine levels found in the substantia nigra and rPut of MPTP-treated monkeys reflect the occurrence of divergent compensatory mechanisms. Specifically, these mechanisms might aim at contrasting the aberrant stimulation of midbrain NMDAR on one hand, while promoting dopamine release from spared striatal dopaminergic terminals on the other hand. Consistent with our assumption, the firing rate of STN glutamatergic neurons projecting to the substantia nigra increases significantly following DA depletion (Hamani et al., 2004), exposing midbrain dopaminergic neurons to potentially neurotoxic concentrations of glutamate (Ambrosi et al., 2014). In this context, the lower availability of the NMDAR co-agonist d-serine may serve to contrast the abnormal activation of this receptor subtype. In contrast, greater levels of d-serine may be required to compensate for the progressive loss of functionality affecting putaminal synapses in the parkinsonian brain since activation of NMDAR on dopaminergic nigrostriatal terminals promotes the release of DA (Chéramy et al., 1998; Krebs et al., 1991; Pittaluga, 2021) and enables synaptic plasticity (Calabresi et al., 1992). The response of MPTPp treatment in mice significantly differed from those in MPTP-treated monkeys, as we found that the levels of both serine enantiomers and the d-serine/total serine ratio were unchanged in the CPu, mPFC, and SNc as compared to controls. Moreover, MPTPp-treated mice did not manifest any variation in the striatal expression of GAPDH and ASCT1. As alterations in d-serine levels were previously reported in the CPu of bilaterally 6-OHDA-lesioned rats (El Arfani et al., 2015), as well as in the midbrain of MPTP-treated monkeys (Nuzzo et al., 2019) and aged C57BL mice acutely subjected to a high dose of MPTP (30 mg/kg) (Li et al., 2009), we assume that an extensive nigrostriatal dopaminergic degeneration is mandatory for altering the trafficking and concentration of serine enantiomers. Hence, the degree of dopaminergic degeneration reported here in mice receiving sub-chronic MPTPp treatment might not have been sufficient to trigger such alterations in serine metabolism and trafficking, as indicated by the mild MPTPp-induced reduction (~27-30%) of TH immunoreactivity in the CPu and SNc, and by the lack of differences in striatal concentration of L-DOPA, DA, HVA, and DOPAC compared to vehicle-treated controls.

In this respect, it is important to highlight that dopaminergic nigrostriatal neurons are highly adaptive and can compensate for the loss of a significant number of this neuronal population by increasing activity and neurotransmitter turnover at striatal synapses (Blesa et al., 2017). These compensatory changes, along with a decrease in dopamine uptake transporter levels (Mosharov et al., 2015), maintain physiological synaptic DA concentration and DA receptor activation as long as a significant number of dopaminergic neurons are spared from neurodegeneration (Perez et al., 2008). Altogether, our data suggest that the increased levels of serine enantiomers observed in the rPut of MPTP-treated monkeys, and in the CPu and CSF of PD patients (Di Maio et al., 2023), may represent a neuroadaptive modification that appears at the symptomatic phase of the disease, aimed at mitigating the dramatic loss of nigrostriatal dopaminergic input.

We argue that the upregulation of serine enantiomers found in both the CPu of PD patients and MPTP-lesioned monkeys represents an endogenous attempt to curb the ongoing neurodegenerative process. In support of this possibility, l-serine supplementation has been shown to counteract the emergence of neuropathies in diabetic mice (Handzlik et al., 2022) and produce remarkable anti-inflammatory, and neuro-protective effects in experimental models of brain injury (Ye et al., 2021), and neurodegenerative diseases, such as ALS (Holeček, 2022), AD (Le Douce et al., 2020), and PD (Delic et al., 2017).

Compelling evidence supports the beneficial effect of NMDAR co-agonists administration in PD treatment. For instance, d-cycloserine, a partial agonist at the glycine site of NMDAR, was reported to ameliorate behavioural deficits and attenuate neuroinflammation, and neurodegeneration in MPTP-treated rats (Ho et al., 2011), and to improve short-term memory in MPTP-treated monkeys (Schneider et al., 2000). Glycine also acts as an NMDAR co-agonist, and, ACPPB, a potent and selective inhibitor of the glycine transporter 1 (GlyT1), was found to elicit the ventral-to-dorsal sprouting of spared striatal dopaminergic terminals as well as significant DA-related behavioural recovery when administrated to unilaterally 6-OHDA-lesioned mice (Schmitz et al., 2013). Antiparkinsonian and anti-dyskinetic effects have been also recently reported in unilaterally 6-OHDA-lesioned rats and MPTP-treated macaques receiving the GlyT1 inhibitors Bitopertin and ALX-5407, respectively (Frouni et al., 2022; Frouni et al., 2021). Additionally, a recent preliminary investigation conducted on severely DA-depleted MPTP-lesioned mice reported that early treatment with d-serine (0.3 mg/kg; i.p.) markedly ameliorated parkinsonian-like motor symptoms, enhanced TH protein content in the midbrain, and increased DA levels in the CPu (Zhang et al., 2021).

In line with the aforementioned studies and in support of d-serine application in clinical practice (Gelfin et al., 2012; Heresco-Levy et al., 2013), our data highlight the safety of exogenous d-serine supplementation, since one month of oral d-serine administration (100 mg/kg) did not activate microglial cells or alter the integrity of nigrostriatal dopaminergic pathway. Nonetheless, further studies on PD animal models mirroring more advanced phases of the disease will be necessary to substantiate the effects of d-serine supplementation in mitigating the emergence of motor and cognitive deficits. This is relevant considering that the current MPTPp treatment regimen was found to induce only subtle motor deficits in mice (Baranyi et al., 2016).

Noteworthy, in the present study, oral d-serine supplementation does not promote dopaminergic reinnervation in the CPu of MPTPp-treated mice, as previously described in 6-OHDA-lesioned mice receiving the Glyt1 blocker ACPPB (Schmitz et al., 2013). This discrepancy might be explained by the different experimental conditions, drugs, and PD animal models used in these studies (Schmitz et al., 2013). Other investigations are warranted to address this key issue and analyse the consequences of d-serine supplementation in different PD animal models under different administration schedules. Indeed, a limitation of the present work arises from the utilization of animal models that involve the administration of a neurotoxin, which fails to replicate all key features of the disease, including the progressive neurodegeneration triggered by the accumulation of alpha-synuclein in the dopaminergic nigrostriatal system. Therefore, other studies utilizing animal models such as rats and monkeys treated with viral vectors expressing the human alpha-synuclein gene or pre-formed alpha-synuclein fibrils are warranted to complement the current observations and establish the extent of dopaminergic degeneration instrumental to induce variation in serine enantiomer levels. A second limitation of the present study pertains to the limited number of monkeys used (*n* = 5/group), as well as the sex of the animals. Indeed, our experiments on monkeys and mice were conducted exclusively on females and males, respectively. In this respect, previous studies evaluating the serum and *post-mortem* hippocampus of patients with AD have highlighted a significant gender effect in the concentration of serine enantiomers as well as in the d-serine/total serine ratio (Maffioli et al., 2022; Piubelli et al., 2021). Therefore, other experiments are mandatory to determine whether similar gender differences can be detected in animal models of PD. Nonetheless, it is important to emphasize that the increase in d-serine and l-serine levels found in the *post-mortem* CPu and CSF of living PD patients did not differ between male and female subjects (Di Maio et al., 2023).

## 5. Conclusion

**Fig. 9.**
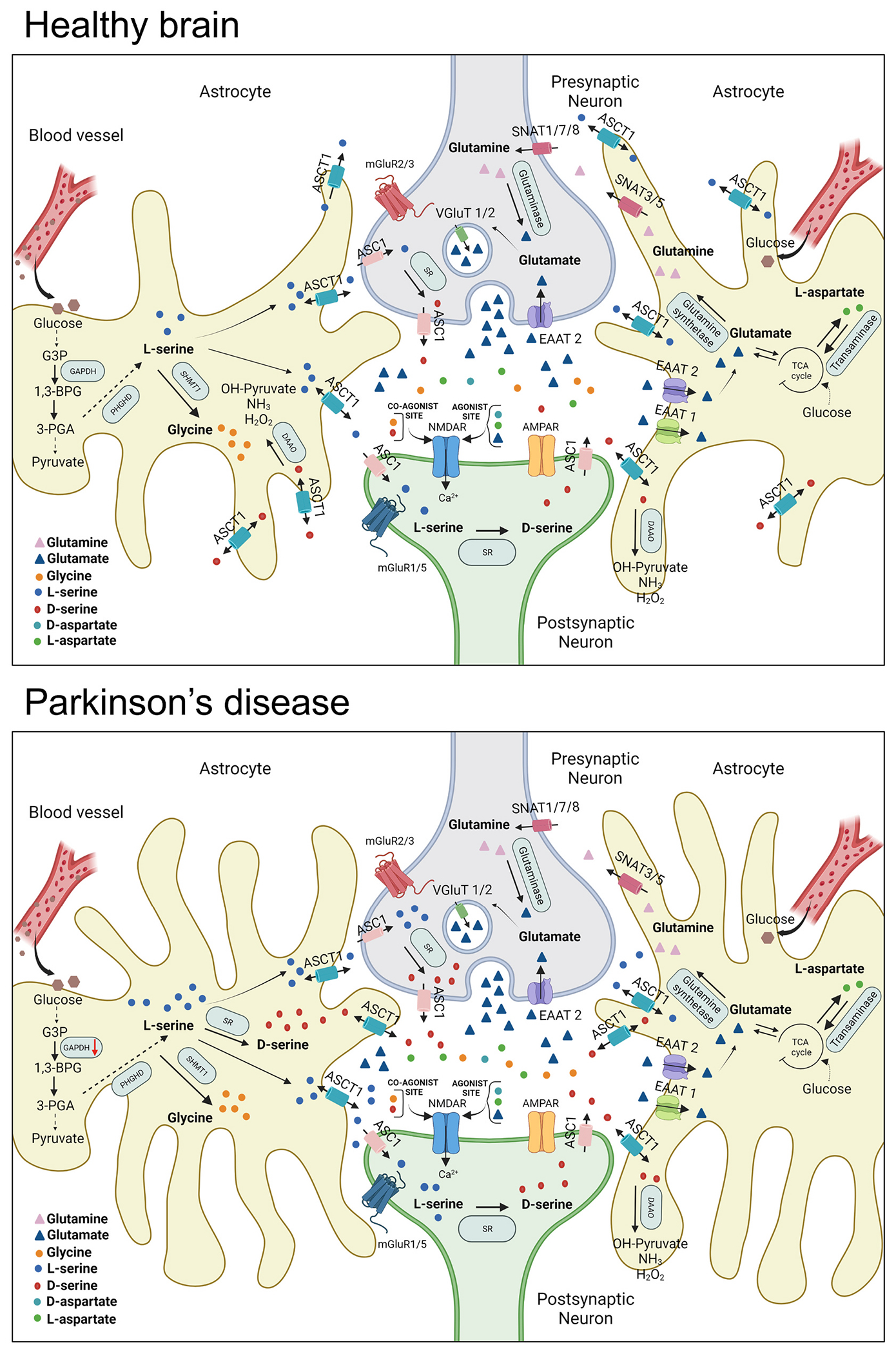
Proposed model illustrating alterations in d-serine and l-serine levels in striatal glutamatergic synapses in healthy and Parkinson’s disease disease. In astrocytes, the biosynthesis of l-serine is a complex enzymatic process that entails the conversion of glucose into the glycolytic intermediate 3-phosphoglycerate (3-PGA), followed by the enzymatic transformation of 3-PGA into l-serine through the phosphorylated pathway (Rabattoni et al., 2023). In the healthy brain (top panel), endogenous l-serine can undergo two pathways: (i) conversion into the NMDAR co-agonist glycine via the serine hydroxymethyltransferase (SHMT1) enzyme, or (ii) transportation across the astrocytic membrane into the pre- and post-synaptic neuronal compartments through the alanine serine cysteine transporter 1 (ASCT1, SLC1A4) and ASC1 (SLC7A10) transporters, located on the astrocytic and neuronal membrane, respectively (Ivanov and Mothet, 2019; Kaplan et al., 2018). Once in neurons, l-serine is enzymatically converted by serine racemase (SR) into d-serine, which is subsequently released into the synaptic cleft by ASC1, thereby enabling NMDAR activation (Douce et al., 2020). Synaptic d-serine is then transported into astrocytes and catabolized by the D-amino acid oxidase (DAAO) enzyme (Krishnan and Billups, 2023). In Parkinson’s disease (bottom panel), the homeostasis of striatal d-serine and l-serine is severely disrupted. In MPTP-treated monkeys and parkinsonian patients (Di Maio et al., 2023), loss of midbrain dopaminergic neurons leads to a significant elevation of both striatal d-serine and l-serine levels, coupled with a reduced expression of the glycolytic enzyme glyceraldehyde-3-phosphate dehydrogenase (GAPDH), and the astrocytic serine transporter, ASCT1. Considering the switch in SR expression and d-serine production from neurons to astrocytes reported under pathological circumstances (Coyle et al., 2020; Wolosker et al., 2016), and the significant striatal astrogliosis found in post-mortem samples from animal model models and parkinsonian patients (Charron et al., 2014), we hypothesize that alterations in astrocytes may represent a key factor contributing to aberrant higher striatal serine enantiomers levels found in PD. Figure legend: The modification in astrocyte morphology across panels reflects variations in astrocytic activation subsequent to dopamine denervation. The downward red arrow signifies a decrease in protein expression. Abbreviations: 1.3-BPG, 1,3-bisphosphoglyceric acid; 3-PGA, 3-phosphoglyceric acid; AMPAR, α-amino-3-hydroxy-5-methyl-4-isoxazolepropionic acid receptor; EAAT, excitatory amino acid transporters; G3P, glycerol-3-phosphate; mGluR, metabotropic glutamate receptor; vGluT, vesicular glutamate transporter. The illustration was created with Biorender.com.

The present work provides compelling evidence in support of our recent clinical study showing altered serine enantiomers metabolism in the post-mortem CPu of parkinsonian brains and in the CSF of de novo living patients (Di Maio et al., 2023). Indeed, we demonstrated that a profound loss of nigrostriatal dopaminergic neurons, as that found in MPTP-treated monkeys but not in MPTPp-treated mice, is required to trigger detectable changes in the striatal concentration of serine enantiomers. Moreover, as illustrated in Fig. 9, the observations indicating a reduced expression of the ubiquitous glycolytic enzyme, GAPDH, and of the astrocytic serine transporter, ASCT1, in the rPut of MPTP-treated monkeys, hold substantial significance in light of the emerging understanding of disrupted energy homeostasis and astrocyte dysfunction in the pathophysiology of PD.

## Supplementary Material

supplemental material 1

supplemental material 2

## Data Availability

The datasets used and/or analysed during the current study are available from the corresponding author upon reasonable request.

## Appendix A. Supplementary data

Supplementary data to this article can be found online at https://doi.org/10.1016/j.nbd.2023.106226.

## Abbreviations:

6-OHDA: 6-hydroxydopamine
AD: Alzheimer’s disease
ALS: Amyotrophic Lateral Sclerosis
ASCT1: alanine cysteine serine transporter 1
CPu: caudate putamen
CSF: cerebrospinal fluid
DA: dopamine
DAAO: D-amino acid oxidase
DOPAC: 3,4-dihydroxyphenylacetic acid
GAPDH: glyceraldehyde-3-phosphate dehydrogenase
GP: globus pallidus
HPLC: high-performance liquid chromatography
HVA: homovanillic acid
IBA1: ionized calcium-binding adaptor molecule 1
IL-1β: interleukin-1β
IQR: interquartile range
L-DOPA: levodopa
mPFC: medial prefrontal cortex
MPTP: 1-methyl-4-phenyl-1,2,3,6-tetrahydropyridine
MPTPp: MPTP-plus-probenecid
NMDAR: ionotropic N-methyl-D-aspartate receptors
PD: Parkinson’s disease
PHGDH: 3-phosphoglycerate dehydrogenase
rPut: rostral putamen
SNc: substantia nigra pars compacta
SR: serine racemase
STN: subthalamic nucleus
TH: tyrosine hydroxylase
WB: western blot
