# Supplementary material for: Perturbation of serine enantiomers homeostasis in the striatum of MPTP-lesioned monkeys and mice reflects the extent of dopaminergic midbrain degeneration
Source: Neurobiol Dis. Author manuscript; Available in PMC 2025 Dec 26. (PMC12741449; doi:10.1016/j.nbd.2023.106226)
Supplement: supplemental material 1 [file NIHMS2124576-supplement-supplemental_material_1.docx]

*Supplementary info for*

***Perturbation of serine enantiomers homeostasis in the striatum of MPTP-lesioned monkeys and mice reflects the extent of dopaminergic midbrain degeneration.***

Marcello Serra^1†^, Anna Di Maio^2, 3†^, Valentina Bassareo^1^, Tommaso Nuzzo^2,3^, Francesco Errico^2, 4^ Federica Servillo^5^, Mario Capasso^2,6^, Pathik Parekh^1^, Qin Li^7, 8^, Marie-Laure Thiolat^9, 10^, Erwan Bezard^7, 8, 9, 10^, Paolo Calabresi^5, 11^, David Sulzer^12^, Manolo Carta^1^, Micaela Morelli^1, 13^, Alessandro Usiello^2, 3^*

^1^Department of Biomedical Sciences, University of Cagliari, Monserrato, Italy;

^2^Laboratory of Translational Neuroscience, CEINGE Biotecnologie Avanzate Francesco Salvatore, Naples, Italy;

^3^Department of Environmental, Biological and Pharmaceutical Science and Technologies, Università degli Studi della Campania "Luigi Vanvitelli", Caserta, Italy;

^4^Department of Agricultural Sciences, University of Naples “Federico II”, Naples, Italy;

^5^Department of Neuroscience, Cattolica Sacro Cuore University, Rome, Italy;

^6^Dipartimento di Medicina Molecolare e Biotecnologie Mediche, Università degli Studi di Napoli Federico II, Via Pansini, 5, 80131 Napoli, Italy;

^7^Motac Neuroscience, UKM15 6WE, Manchester, United Kingdom;

^8^Institute of Lab Animal Sciences, China Academy of Medical Sciences, Beijing, China;

^9^Université de Bordeaux, Institut des Maladies Neurodégénératives, Bordeaux, France;

^10^Centre National de la Recherche Scientifique Unité Mixte de Recherche 5293, Institut des Maladies Neurodégénératives, Bordeaux, France;

^11^Neurologia, Policlinico Universitario A. Gemelli, IRCCS, Rome, Italy

^12^Departments of Psychiatry, Neurology, Pharmacology, Columbia University Irving Medical Center, Division of Molecular Therapeutics, New York State Psychiatric Institute, New York, NY 10032, USA;

^13^National Research Council of Italy, Institute of Neuroscience, Cagliari, Italy.

†These authors contributed equally to this work.

* Corresponding Author:

Alessandro Usiello, Ph.D.: Department of Environmental, Biological and Pharmaceutical Sciences and Technologies, University of Campania “Luigi Vanvitelli”, Via A. Vivaldi, 43, 81100 Caserta, Italy, and CEINGE Biotecnologie Avanzate, Naples, Italy; Phone: +39 0813737879, email: alessandro.usiello@unicampania.it;

**Supplemental Figures**

**
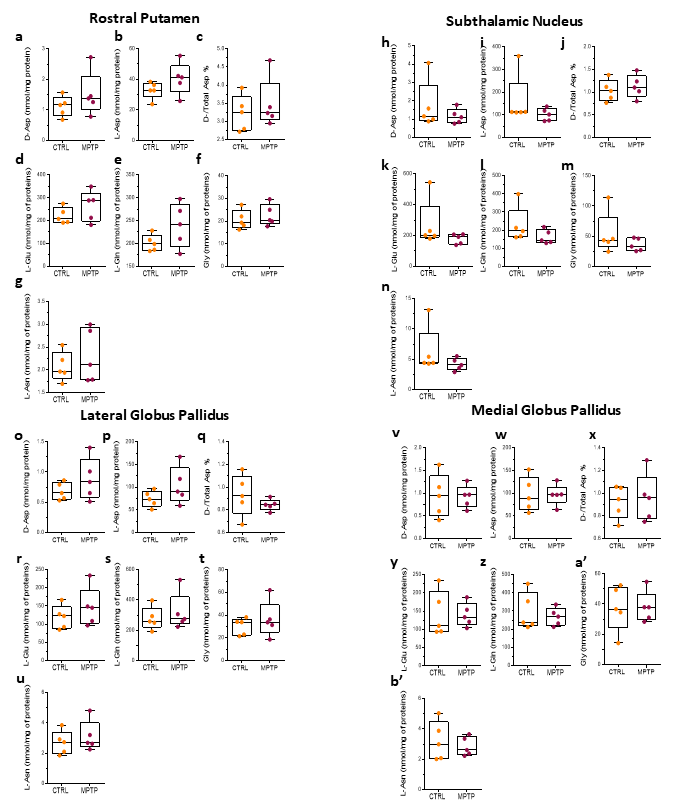
**

**Supplemental Figure S1.** Brain levels of aspartate enantiomers, L-glutamate, L-glutamine, glycine, L-asparagine, and the D-total/aspartate ratio in healthy and MPTP-treated monkeys. (**a-b’**) Box plots indicating the concentration of aspartate enantiomers, L-glutamate, L-glutamine, glycine, L-asparagine, and the D-/total aspartate ratio in the rostral putamen (rPut) (**a-g**; p>0.05), subthalamic nucleus (STN) (**h-n;** p>0.05), lateral globus pallidus (GP) (**o-u;** p>0.05), and medial GP (**v-b’;** p>0.05) of control and MPTP-treated monkeys. Box plots indicate the top and bottom quartiles; whiskers refer to top and bottom 90%. Abbreviations: MPTP, 1-methyl-4-phenyl-1.2.3.6-tetrahydropyridine; STN, subthalamic nucleus; D-/L-Asp, D-/L-aspartate; L-Glu, L-glutamate; L-Gln, L-glutamine; Gly, glycine; L-Asn, L-asparagine. Colour legend: orange. CTRL; red. MPTP.


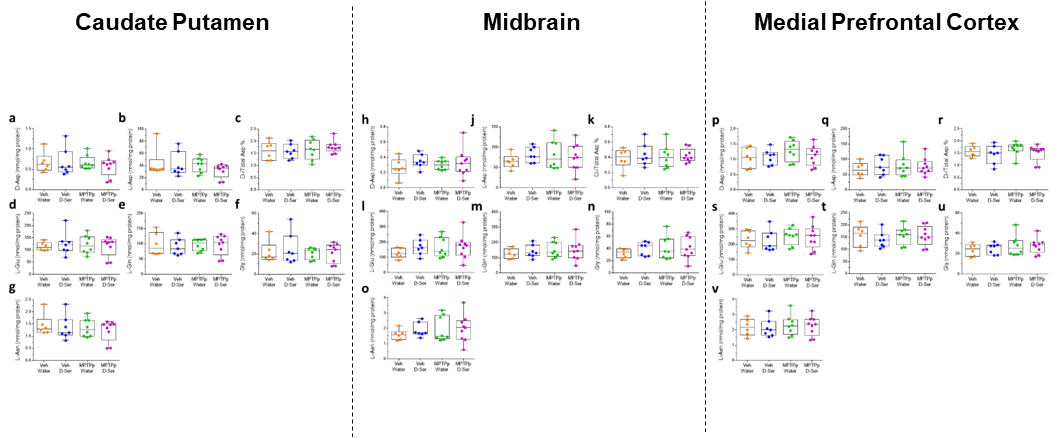


**Supplemental Figure S2.** Brain levels of aspartate enantiomers, L-glutamate, L-glutamine, glycine, L-asparagine, and the D-total/aspartate ratio in vehicle- and MPTPp-treated mice following oral D-serine supplementation. Representative box plots indicating the concentration of aspartate enantiomers, L-glutamate, L-glutamine, glycine, L-asparagine, and the D-/total aspartate ratio in the caudate putamen (**a-g**; p>0.05), midbrain (**h-o**; p>0.05), and medial prefrontal cortex (**p-v**; p>0.05) of Veh/Water, Veh/D-ser, MPTPp/Water, and MPTPp/D-ser treated-mice. Box plots indicate the top and bottom quartiles; whiskers refer to top and bottom 90%. N=6-9 mice/treatment. Abbreviations: D-ser, D-serine; MPTP, 1-methyl-4-phenyl-1,2,3,6-tetrahydropyridine; MPTPp, MPTP+probenecid; Veh, Vehicle; D-/L-Asp, D-/L-aspartate; L-Glu, L-glutamate; L-Gln, L-glutamine; Gly, glycine; L-Asn, L-asparagine. Colour legend: orange, Veh/Water; blue, Veh/D-ser; green, MPTPp/Water; violet, MPTPp/D-ser
