# Supplementary material for: Perturbation of serine enantiomers homeostasis in the striatum of MPTP-lesioned monkeys and mice reflects the extent of dopaminergic midbrain degeneration
Source: Neurobiol Dis. Author manuscript; Available in PMC 2025 Dec 26. (PMC12741449; doi:10.1016/j.nbd.2023.106226)
Supplement: supplemental material 2 [file NIHMS2124576-supplement-supplemental_material_2.docx]

*Supplemental material for*

***Perturbation of serine enantiomers homeostasis in the striatum of MPTP-lesioned monkeys and mice reflects the extent of dopaminergic midbrain degeneration.***

Marcello Serra^1†^, Anna Di Maio^2, 3†^, Valentina Bassareo^1^, Tommaso Nuzzo^2,3^, Francesco Errico^2, 4^ Federica Servillo^5^, Mario Capasso^2,6^, Pathik Parekh^1^, Qin Li^7, 8^, Marie-Laure Thiolat^9, 10^, Erwan Bezard^7, 8, 9, 10^, Paolo Calabresi^5, 11^, David Sulzer^12^, Manolo Carta^1^, Micaela Morelli^1, 13^, Alessandro Usiello^2, 3^*

^1^Department of Biomedical Sciences, University of Cagliari, Monserrato, Italy;

^2^Laboratory of Translational Neuroscience, CEINGE Biotecnologie Avanzate Francesco Salvatore, Naples, Italy;

^3^Department of Environmental, Biological and Pharmaceutical Science and Technologies, Università degli Studi della Campania "Luigi Vanvitelli", Caserta, Italy;

^4^Department of Agricultural Sciences, University of Naples “Federico II”, Naples, Italy;

^5^Department of Neuroscience, Cattolica Sacro Cuore University, Rome, Italy;

^6^Dipartimento di Medicina Molecolare e Biotecnologie Mediche, Università degli Studi di Napoli Federico II, Via Pansini, 5, 80131 Napoli, Italy;

^7^Motac Neuroscience, UKM15 6WE, Manchester, United Kingdom;

^8^Institute of Lab Animal Sciences, China Academy of Medical Sciences, Beijing, China;

^9^Université de Bordeaux, Institut des Maladies Neurodégénératives, Bordeaux, France;

^10^Centre National de la Recherche Scientifique Unité Mixte de Recherche 5293, Institut des Maladies Neurodégénératives, Bordeaux, France;

^11^Neurologia, Policlinico Universitario A. Gemelli, IRCCS, Rome, Italy

^12^Departments of Psychiatry, Neurology, Pharmacology, Columbia University Irving Medical Center, Division of Molecular Therapeutics, New York State Psychiatric Institute, New York, NY 10032, USA;

^13^National Research Council of Italy, Institute of Neuroscience, Cagliari, Italy.

†These authors contributed equally to this work.

* Corresponding Author:

Alessandro Usiello, Ph.D.: Department of Environmental, Biological and Pharmaceutical Sciences and Technologies, University of Campania “Luigi Vanvitelli”, Via A. Vivaldi, 43, 81100 Caserta, Italy, and CEINGE Biotecnologie Avanzate, Naples, Italy; Phone: +39 0813737879, email: alessandro.usiello@unicampania.it;


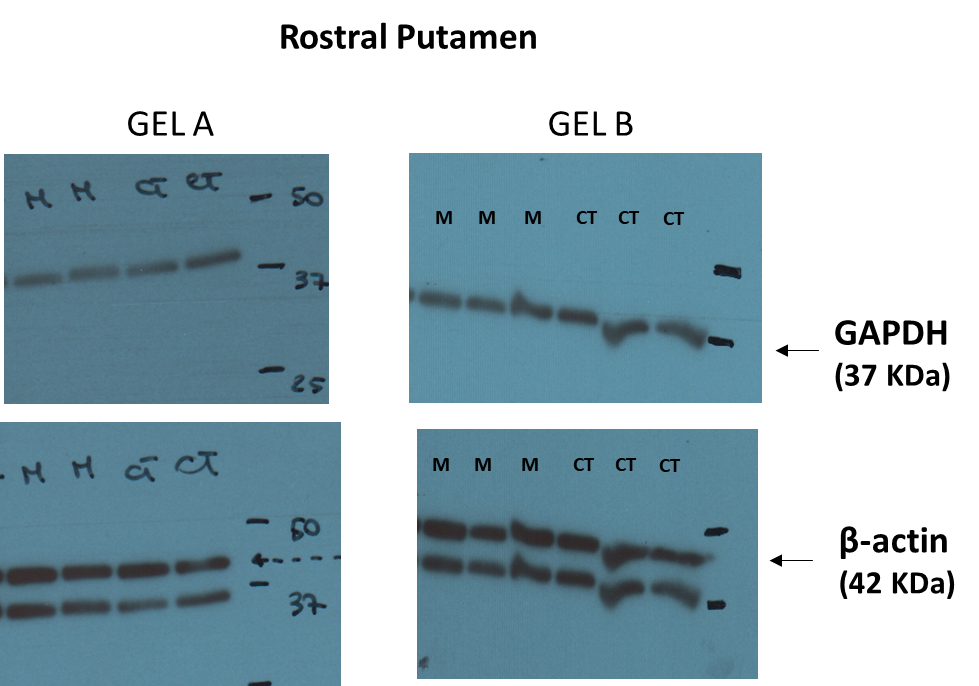


**Figure 1.** Images of the original western blots showing the immunodensity for the glyceraldehyde-3-phosphate dehydrogenase and β-actin in lysate samples obtained from the *post-mortem* rostral putamen of controls and MPTP-treated monkeys. Legend: CT, control; GAPDH, glyceraldehyde-3-phosphate dehydrogenase.


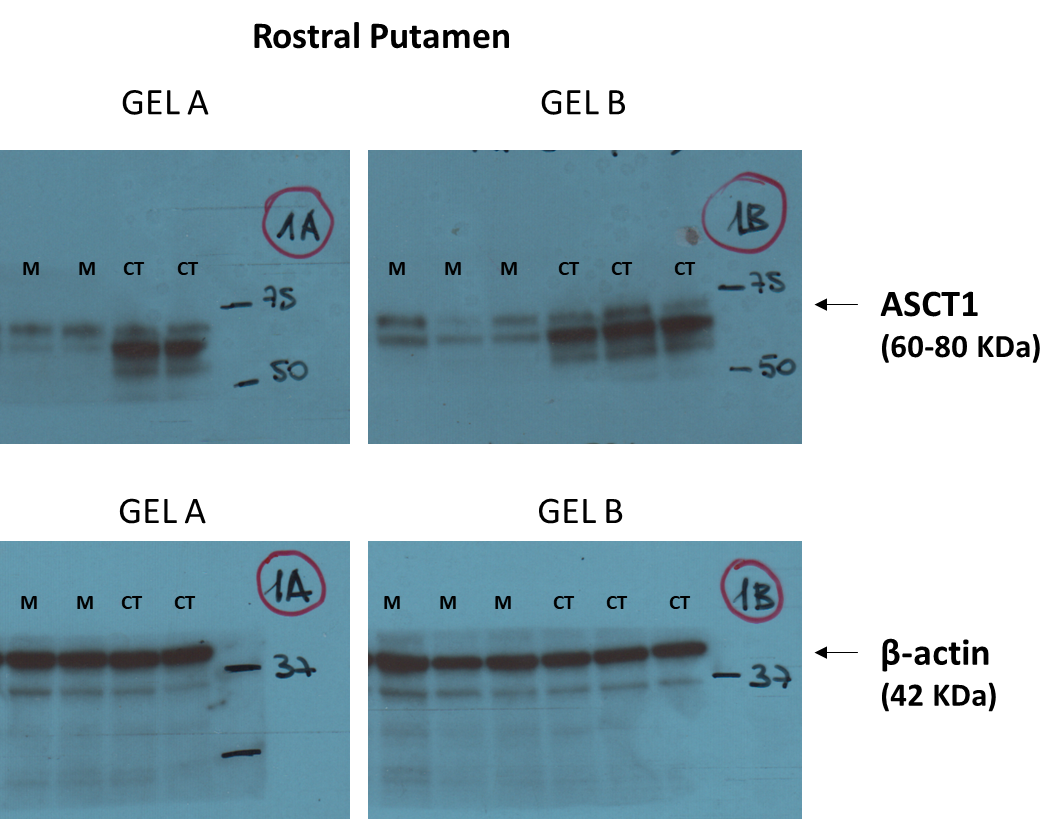


**Figure 2.** Images of the original western blots showing the immunodensity for the alanine serine cysteine transporter 1 (ASCT1) and β-actin in lysate samples obtained from the *post-mortem* rostral putamen of controls and MPTP-treated monkeys. Legend: CT, control; GAPDH, glyceraldehyde-3-phosphate dehydrogenase.


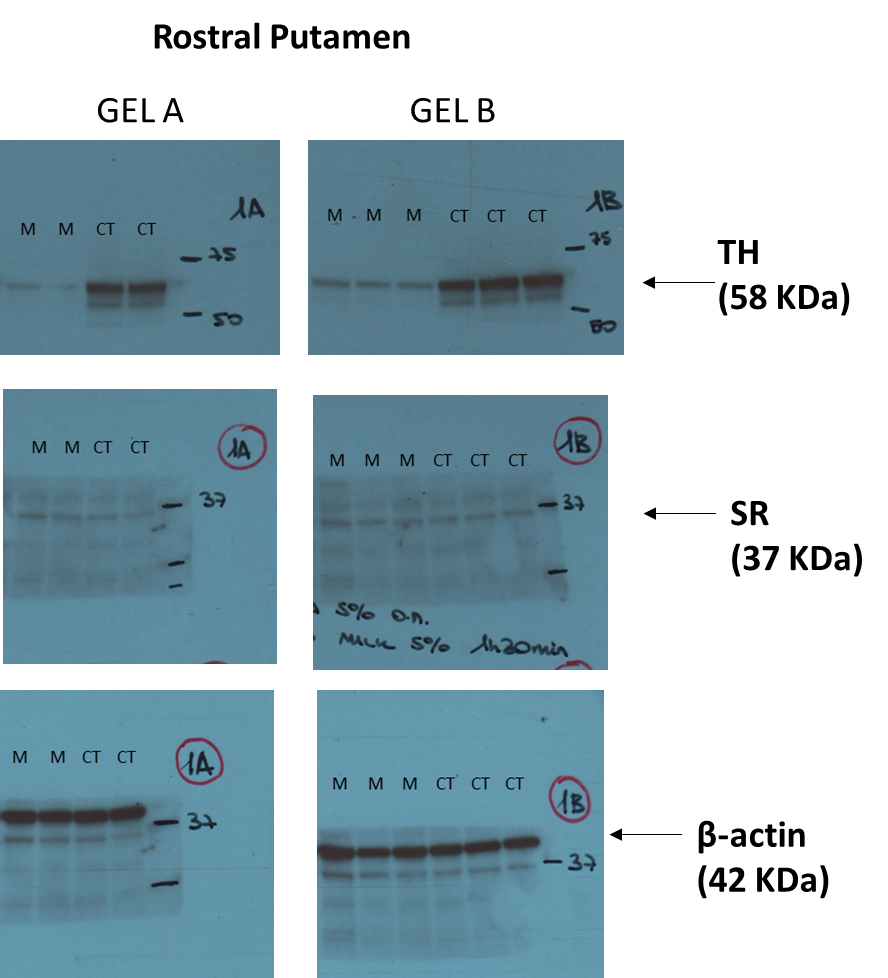


**Figure 3.** Images of the original western blots showing the immunodensity for the tyrosine hydroxylase, serine racemase, and β-actin in lysate samples obtained from the *post-mortem* rostral putamen of controls and MPTP-treated monkeys. Legend: CT, control; GAPDH, glyceraldehyde-3-phosphate dehydrogenase.


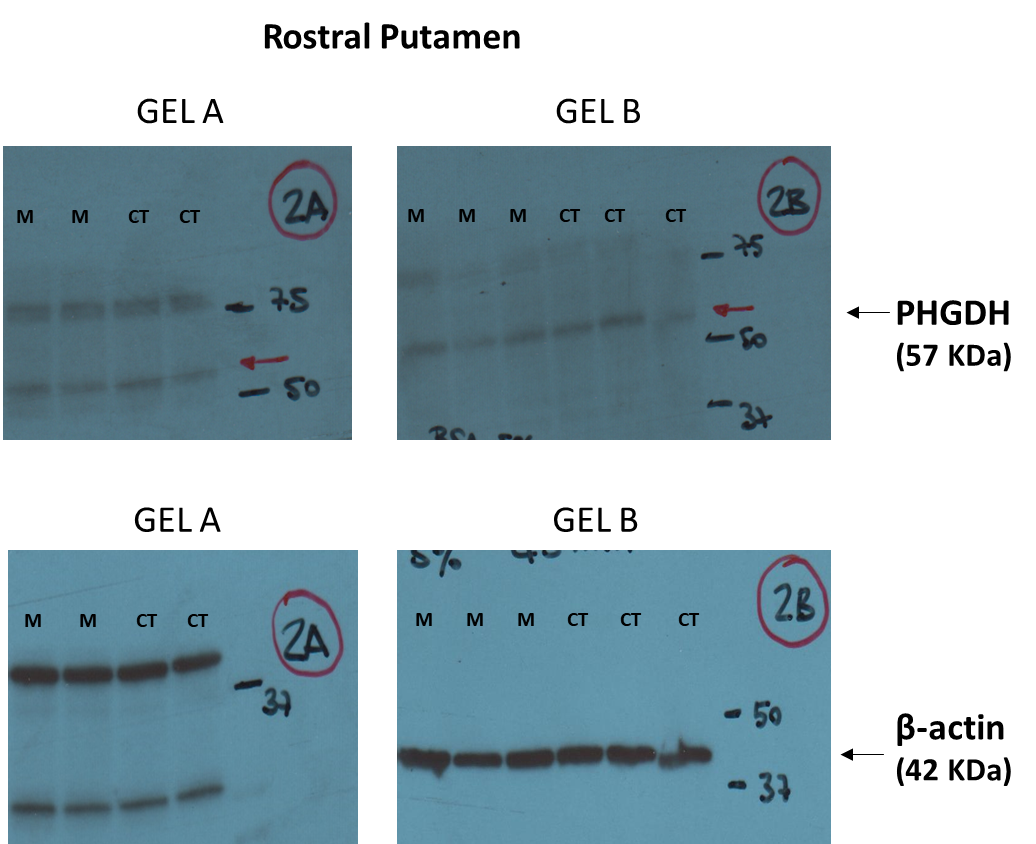


**Figure 4.** Images of the original western blots showing the immunodensity for 3-phosphoglycerate dehydrogenase (PHGDH), and β-actin in lysate samples obtained from the *post-mortem* rostral putamen of controls and MPTP-treated monkeys. Legend: CT, control; GAPDH, glyceraldehyde-3-phosphate dehydrogenase.


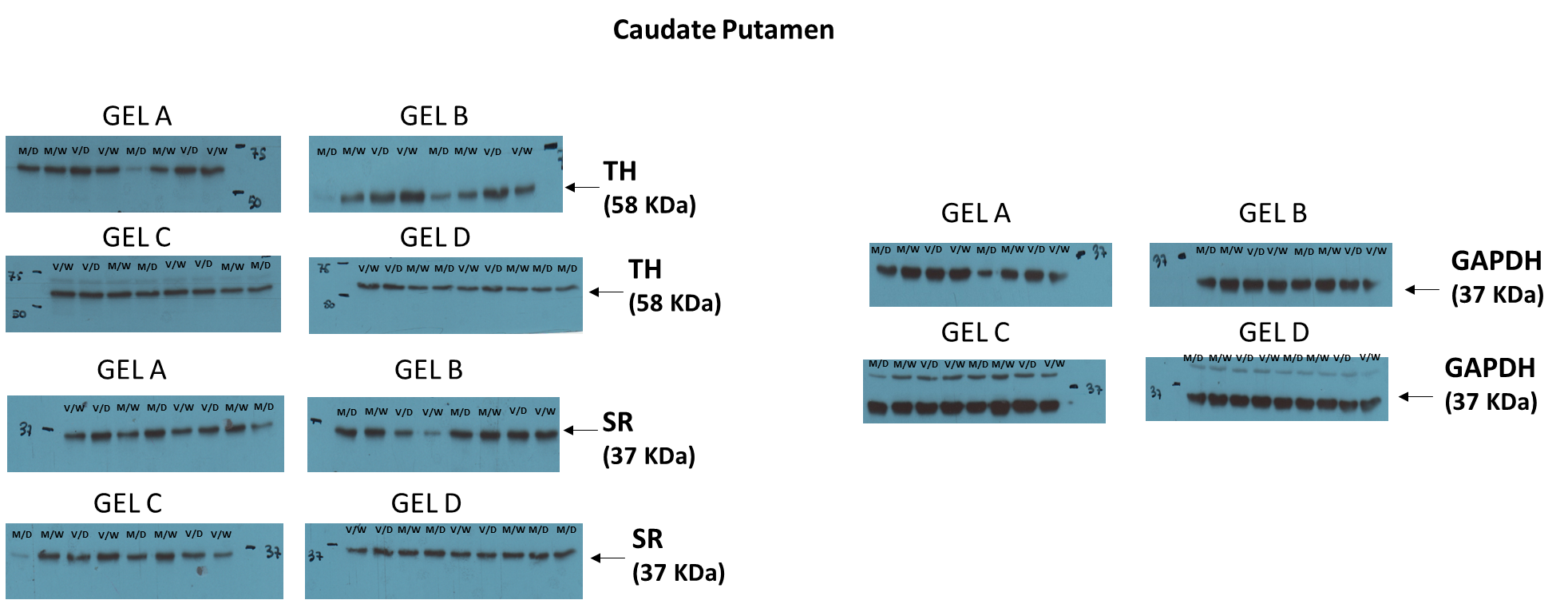


**Figure 5.** Images of the original western blots showing the immunodensity for the tyrosine hydroxylase, serine racemase, and glyceraldehyde-3-phosphate dehydrogenase (GAPDH) in lysate samples obtained from the *post-mortem* caudate putamen of mice treated with either vehicle/water (V/W), vehicle/D-serine (V/D), MPTPp/water (M/W), or MPTPp/D-serine (M/D). Legend: SR, serine racemase, TH, tyrosine hydroxylase, GAPDH, glyceraldehyde-3-phosphate dehydrogenase


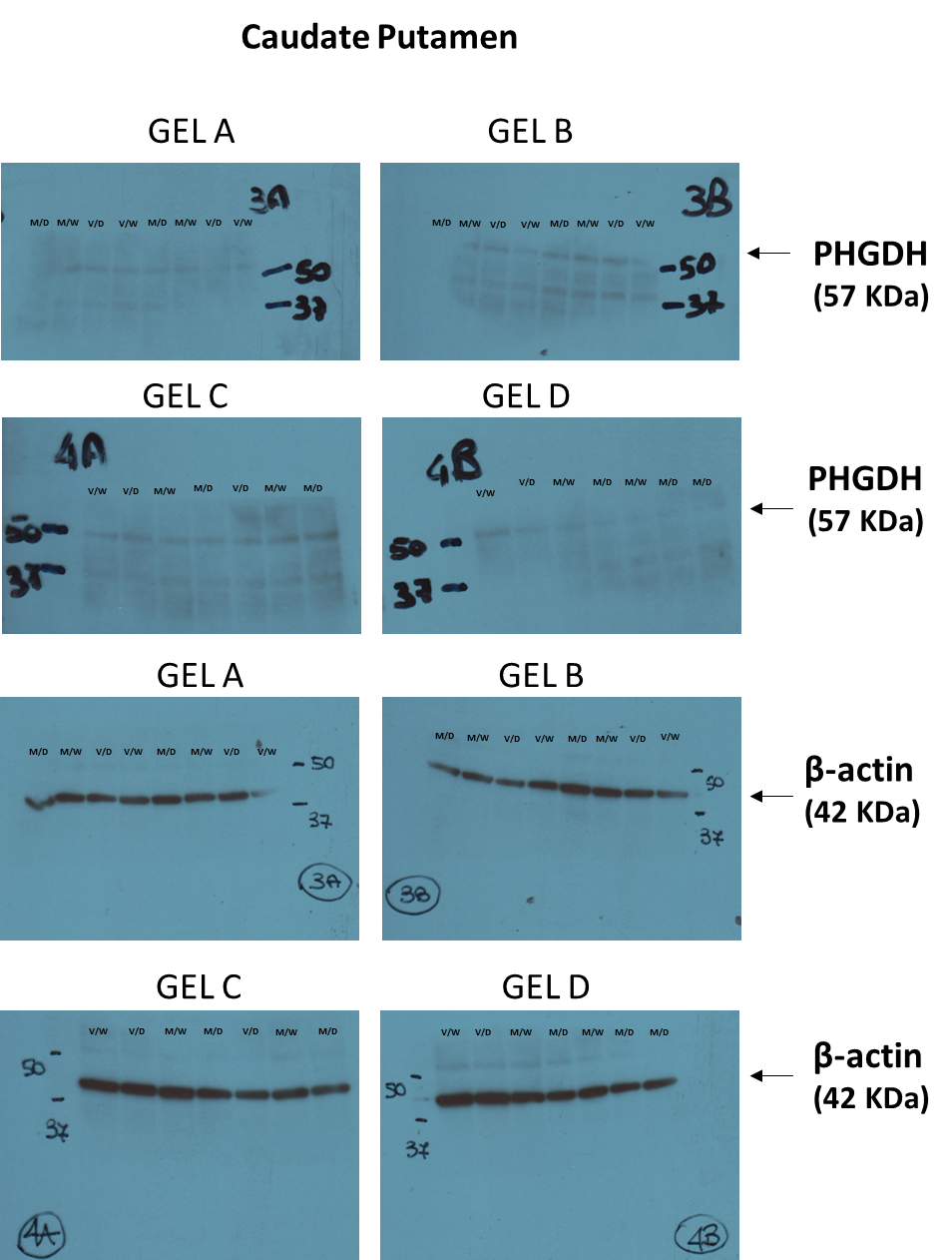


**Figure 6.** Images of the original western blots showing the immunodensity for the 3-phosphoglycerate dehydrogenase (PHGDH), and β-actin in lysate samples obtained from the *post-mortem* caudate putamen of mice treated with either vehicle/water (V/W), vehicle/D-serine (V/D), MPTPp/water (M/W), or MPTPp/D-serine (M/D). Legend: SR, serine racemase, TH, tyrosine hydroxylase, GAPDH, glyceraldehyde-3-phosphate dehydrogenase.


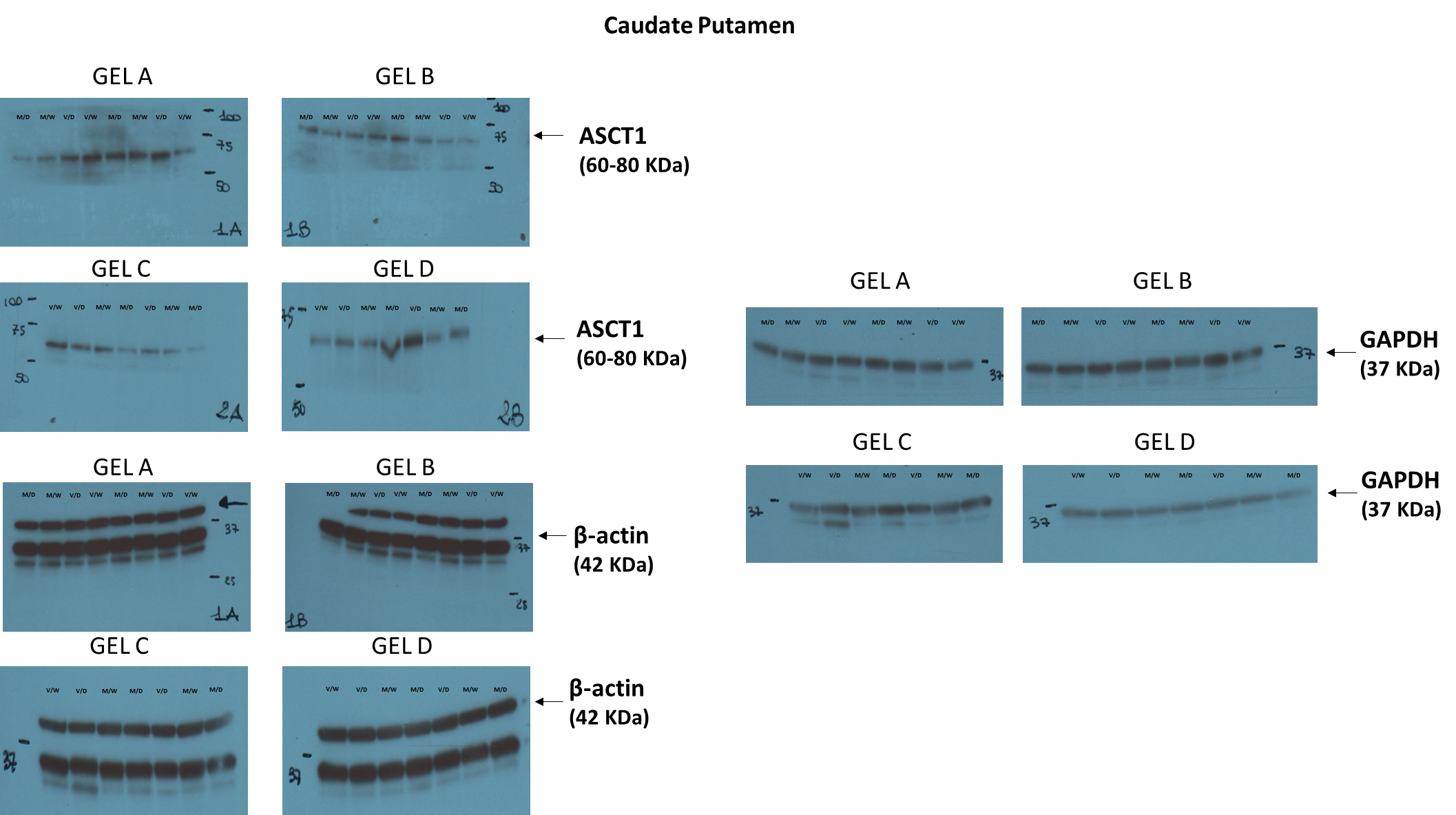


**Figure 7.** Images of the original western blots showing the immunodensity for the alanine serine cysteine transporter 1 (ASCT1), glyceraldehyde-3-phosphate dehydrogenase (GAPDH), and β-actin in lysate samples obtained from the *post-mortem* caudate putamen of mice treated with either vehicle/water (V/W), vehicle/D-serine (V/D), MPTPp/water (M/W), or MPTPp/D-serine (M/D). Legend: SR, serine racemase, TH, tyrosine hydroxylase, GAPDH, glyceraldehyde-3-phosphate dehydrogenase.
